# Testing a key assumption in animal communication: between-individual variation in female visual systems alters perception of male signals

**DOI:** 10.1242/bio.028282

**Published:** 2017-12-15

**Authors:** Kelly L. Ronald, Amanda L. Ensminger, Matthew D. Shawkey, Jeffrey R. Lucas, Esteban Fernández-Juricic

**Affiliations:** 1 Indiana University, Department of Biology, Jordan Hall, 1001 E 3rd Street, Bloomington, IN 47405, USA; 2 Purdue University, Department of Biological Sciences, Lilly Hall, 915 West State Street, West Lafayette, IN 47907, USA; 3 Morningside College, Biology Department, 1501 Morningside Avenue, Sioux City, IA 51106, USA; 4 Evolution and Optics of Nanostructure Group, Department of Biology, University of Ghent, Ledeganckstraat 35, Ghent 9000, Belgium

**Keywords:** Individual variation, Visual perceptual models, Chromatic contrast

## Abstract

Variation in male signal production has been extensively studied because of its relevance to animal communication and sexual selection. Although we now know much about the mechanisms that can lead to variation between males in the properties of their signals, there is still a general assumption that there is little variation in terms of how females process these male signals. Variation between females in signal processing may lead to variation between females in how they rank individual males, meaning that one single signal may not be universally attractive to all females. We tested this assumption in a group of female wild-caught brown-headed cowbirds (*Molothrus ater*), a species that uses a male visual signal (e.g. a wingspread display) to make its mate-choice decisions. We found that females varied in two key parameters of their visual sensory systems related to chromatic and achromatic vision: cone densities (both total and proportions) and cone oil droplet absorbance. Using visual chromatic and achromatic contrast modeling, we then found that this between-individual variation in visual physiology leads to significant between-individual differences in how females perceive chromatic and achromatic male signals. These differences may lead to variation in female preferences for male visual signals, which would provide a potential mechanism for explaining individual differences in mate-choice behavior.

## Abstract (precis)

**Summary:** Animal communication studies often assume receiver perception is equal across individuals; we found females vary in their visual physiology and perception of male signals which could influence their mating decisions.

## INTRODUCTION

Evolution of male traits via sexual selection requires variation in male signals (Darwin, 1859) and has been extensively documented (Andersson, 1994). Interestingly, recent evidence also suggests there is variation between females in their preference for these male traits (Jennions and Petrie, 1997; Ronald et al., 2012; Edward, 2015; Ah-King and Gowaty, 2016). Between-individual variation in female mate choice could potentially be the result of variation in female sensory perception of male signals (Ronald et al., 2012). However, a prevalent assumption in animal communication is that variation in female sensory perception of male signals is negligible between individual females (Johnstone, 1994; Bateson and Healy, 2005). This implies that female responses are expected to be a direct function of male signal quality, independent of the filtering properties of the female sensory system. This assumption could prevent us from better understanding the evolution of male mating signals.

However, at least conceptually, the complexity of the sensory systems and the signaling environments they evolved in may lead to between-individual variation in signal processing (Dangles et al., 2009; Miller and Bee, 2012; Ronald et al., 2012). In fact, there is some empirical evidence supporting between-individual variation in some sensory traits (Mollon et al., 1984; Fuller et al., 2004; Henry and Lucas, 2010; Ensminger and Fernández-Juricic, 2014; Knott et al., 2017). Nevertheless, no studies to date have examined whether individual variation in sensory filtering capacity exists within a single sex (e.g. females) and whether it could lead to variation in perception. If females perceive male signals differently, it may change the effort that they are willing to invest in sampling potential mates (i.e. choosiness) and/or their overall ranking of potential mates (i.e. preference function) (Jennions and Petrie, 1997; Wagner, 1998). Together, these could influence the overall selection on a particular male trait (Ronald et al., 2012).

The goals of this study were to (1) test the assumption that females have negligible between-individual differences in sensory filtering capacity, and (2) examine whether between-individual variation in sensory filtering capacity could affect the perception of male signals. This study is a critical first step to understanding whether differences in sensory biology can lead to variation in mating preferences and selection for male traits. We used brown-headed cowbird (*Molothrus ater*) females, which actively make mate-choice decisions based on both the quality of the male song and his visual ‘wing-spread’ display (O'Loghlen and Rothstein, 2010; Rothstein et al., 1988; Yokel and Rothstein, 1991). Male cowbirds have two colored-feather regions: their melanin-based brown head, and their structurally based iridescent body coloration that shimmers black/green to humans (McGraw et al., 2002). During courtship, male cowbirds will direct their wing-spread displays towards a female, typically from a short distance (<1 m, Rothstein et al., 1988). Then, while singing, a male will puff up his iridescent breast and body feathers, spread and pump his wings, and end the display in a bow (O'Loghlen and Rothstein, 2010; Rothstein et al., 1988).

On each individual female, we measured two visual traits that have an important role in chromatic and achromatic vision: (1) the density of cones (total and individual cone densities and the proportions of each cone type), and (2) the absorbance properties of cone oil droplets. (1) The density of cones has been implicated in visual spatial resolution (i.e. the higher the cone density, the higher the visual acuity) (Williams and Coletta, 1987; Pettigrew et al., 1988). Differences in cone density could affect the ability of females to distinguish subtle differences in the male visual displays (e.g. degree of breast feather puffing, or the extent of the wingspread). Additionally, the relative density of cones can influence the noise in each photoreceptor channel, such that lower relative cone densities will lead to higher noise and lower chromatic or achromatic discrimination abilities (Vorobyev and Osorio, 1998; Goldsmith and Butler, 2003). Indeed, a recent study found that relative photoreceptor densities were an important factor to determining chromatic contrast values using tetrahedral visual models (Bitton et al., 2017). (2) Birds have oil droplets, small organelles filled with carotenoids, in their cones (Johnston and Hudson, 1976). Oil droplets have an important function in avian color vision because they selectively absorb certain wavelengths of light and consequently shift the spectral sensitivity of visual pigments (Goldsmith, 1984), potentially enhancing hue discrimination (Vorobyev et al., 1998).

We quantified cone densities (and their proportions) and oil droplet absorbance for each individual female. This allowed us to model a female's perception of different male visual signals using a widely accepted model in the visual ecology literature (i.e. photon catch photoreceptor noise-limited model; Vorobyev and Osorio, 1998). The model outputs visual contrast values (chromatic and achromatic), which indicate how much an object (e.g. male visual signal) stands out from the visual background under specific ambient light conditions (Endler, 1990). We estimated chromatic and achromatic contrast for each sampled female, enabling us to predict the conspicuousness of a given male signal from the perspective of each individual female.

## RESULTS

### Cone densities and cone proportions

**Fig. 1.**
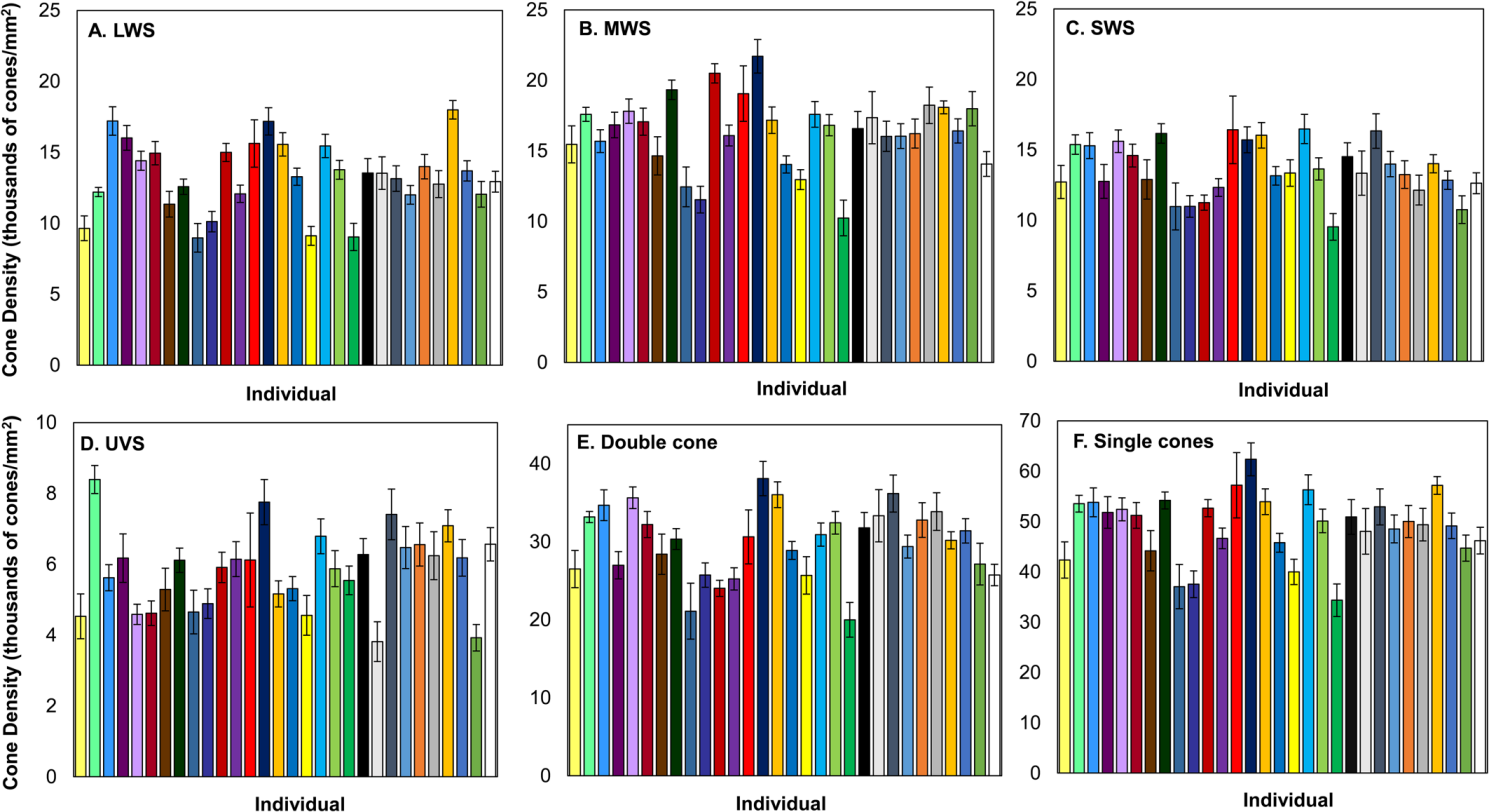
**Variation in cone density across individuals.** *N*=30 for each cone type. LWS (A), MWS (B), SWS (C), and UVS (D), the double cone (E), and all single cones compiled together (F). Error bars are standard error of the mean values.

**Fig. 2.**
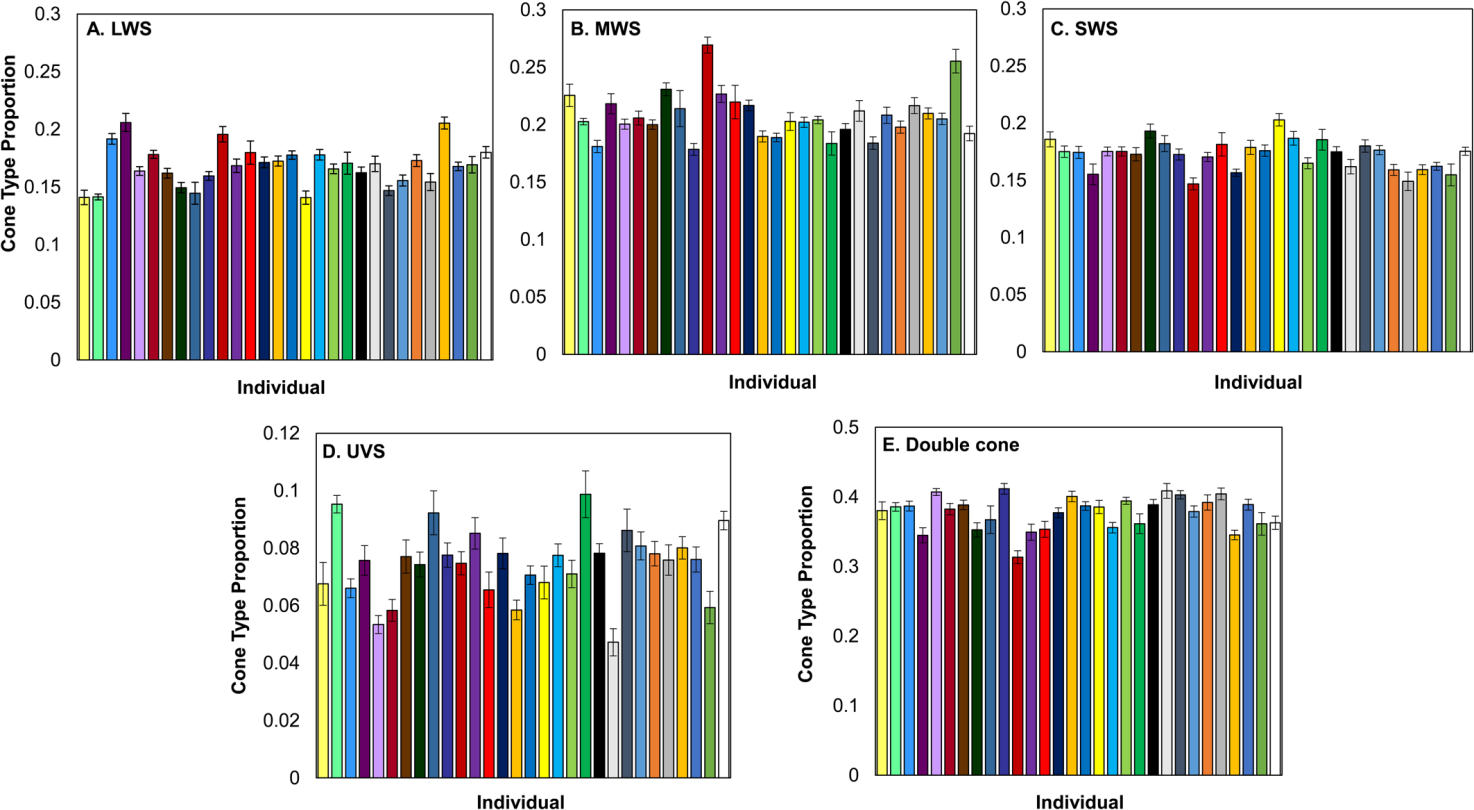
**Variation in cone type proportions across individuals.** *N*=30 for each cone type. LWS (A), MWS (B), SWS (C), and UVS (D), the double cone (E). Error bars are standard error of the mean values.

Females had an average of 79,087.26±1819.79 cones per mm^2^ (100.00% of all cones), 5866.66±194.38 cones per mm^2^ ultra-violet-sensitive (UVS) cones (7.42% of all cones), 13,661.93±350.68 cones per mm^2^ short-wavelength-sensitive (SWS) cones (17.26% of all cones), 16,337.32±448.41 cones per mm^2^ medium-wavelength-sensitive (MWS) cones (20.65% of all cones), 13,389.25±436.61 cones per mm^2^ long-wavelength-sensitive (LWS) cones (16.92% of all cones), and 29,832.09±781.97 cones per mm^2^ double cones (37.72% of all cones). We found significant between-individual variation in the density and the proportion of UVS, SWS, MWS, LWS and double cones (Table 1, Figs 1 and 2). The individual with the highest density of all cones had 44% higher density than the individual with the lowest density (Fig. 1). Overall, individual variation in cone density was the most pronounced in LWS cones, followed by MWS, UVS, double and SWS cones (Table 1). Variation in cone type proportions were similar, although MWS rather than LWS showed the highest variability, followed then by UVS, double, and SWS cones (Fig. 2, Table 1). We found no evidence for a significant association between cone densities and eye axial size or length of time in the lab (see Table 2). The number of days in lab significantly affected the proportion of SWS cones with the proportion of cones decreasing as the time in lab increased (β=−0.0002±0.00006) (Table 2).

**Table 1.**
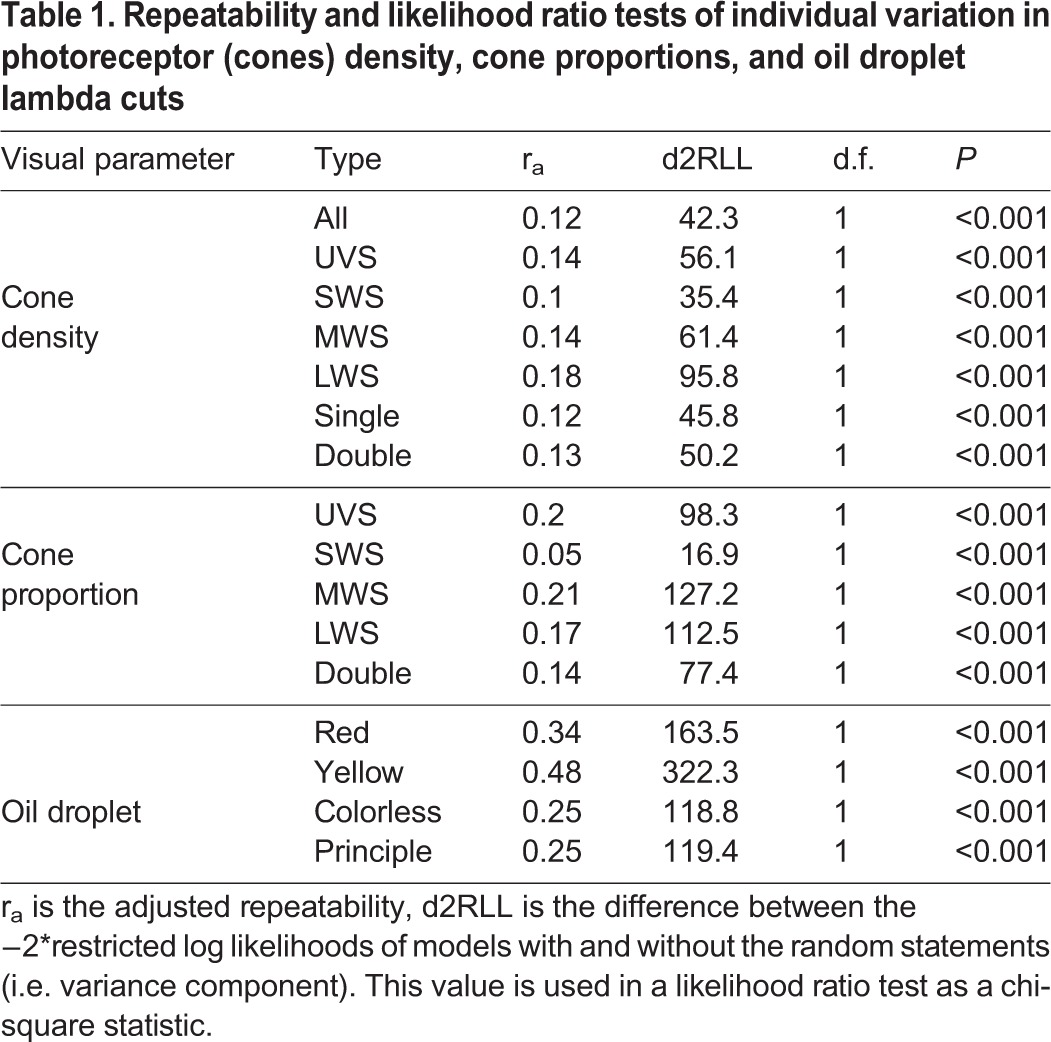
**Repeatability and likelihood ratio tests of individual variation in photoreceptor (cones) density, cone proportions, and oil droplet lambda cuts**

**Table 2.**
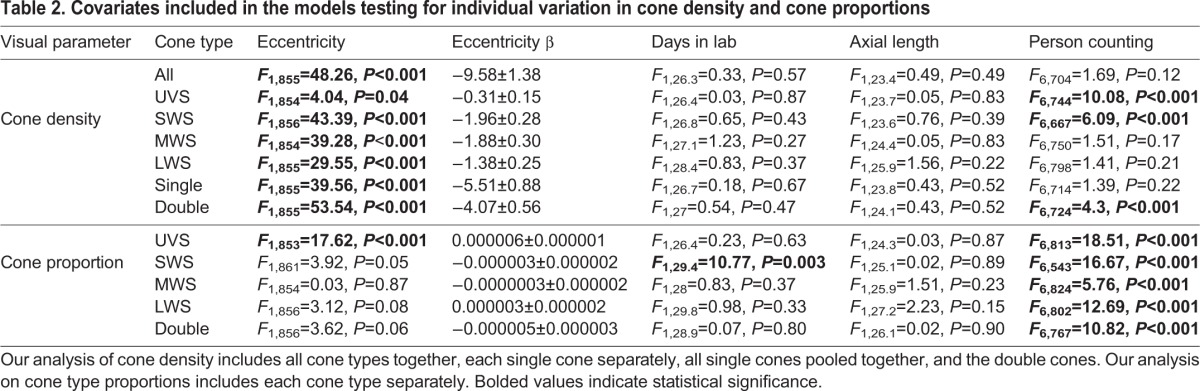
**Covariates included in the models testing for individual variation in cone density and cone proportions**

As expected, we found that all cone type densities significantly decreased with an increase in eccentricity (i.e. distance from the fovea) (Table 2). We also found that the proportion of UVS cones, but no other cone type, significantly increased as distance from the fovea increased (Table 2). Moreover, we found that individual counter identity (i.e. the observer whom calculated cone density) also significantly explained a proportion of the variation in UVS, SWS, and double cone density and proportion values.

### Cone oil droplet lambda cuts

**Fig. 3.**
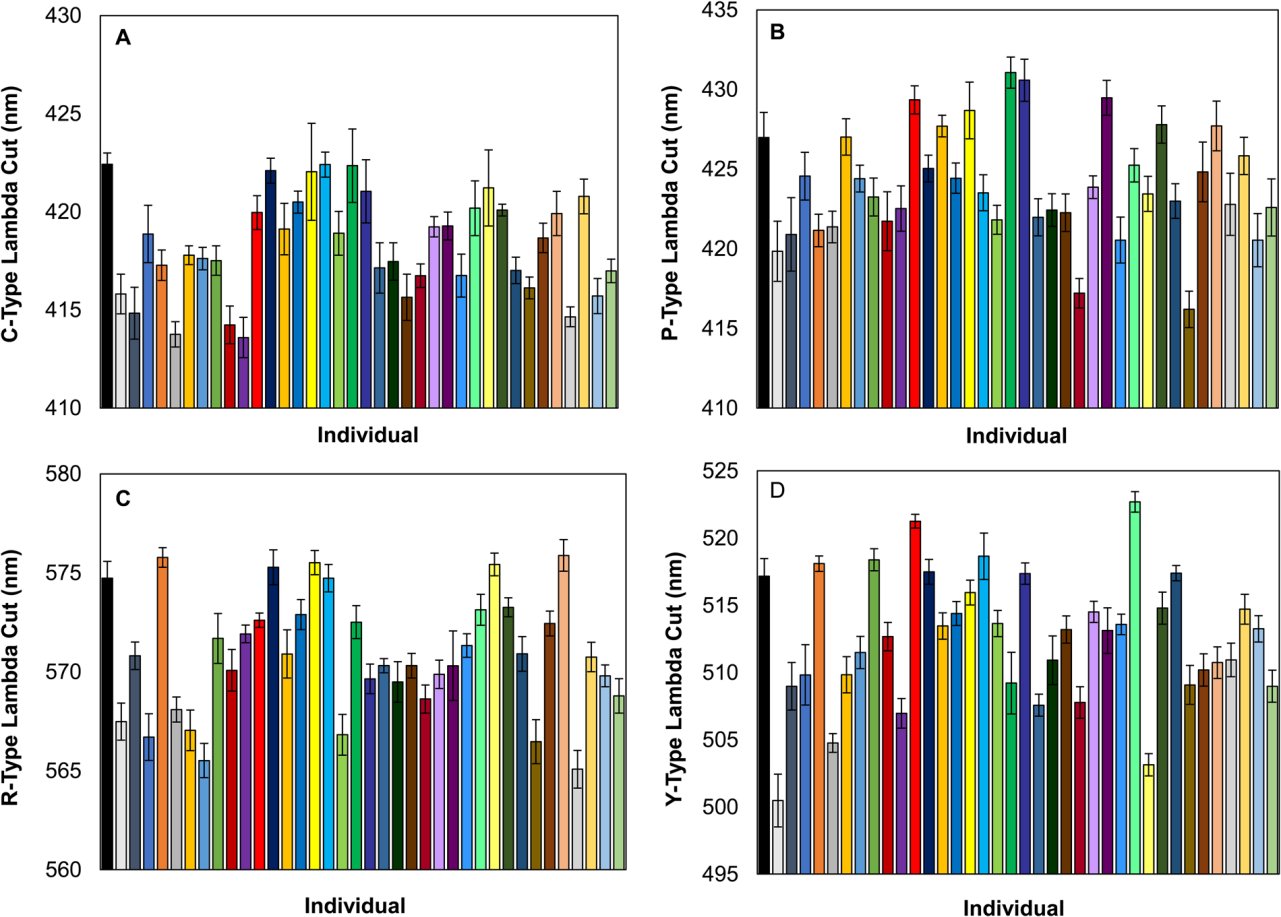
**Variation in cone oil droplet absorbance (λ_cut_) across individuals.** *N*=38. Lambda cuts values for SWS C-type oil droplet (A), the double cone P-type (B), the LWS R-type (C), and the MWS Y-type oil droplet (D). Error bars are standard error of the mean values.

Females had the following average values of λ_cut_: C-type oil droplet (SWS cone), 418.3±0.47 nm; Y-type oil droplet (MWS cone), 512.54±0.88 nm; R-type oil droplet (LWS cone), 570.86±0.55 nm; and P-type oil droplet (double cone), 424.05±0.63 nm. We found significant between-individual variation in the absorbance (λ_cut_) of each oil droplet type (Table 1, Fig. 3). Individual variation was the most pronounced in the Y-type oil droplet (MWS cone), with average λ_cut_ values ranging from 504 nm to 517 nm, followed by the R-type (LWS cone), P-type (double cone), and C-type (SWS) oil droplets (Table 1). We did not find any significant association between oil droplet λ_cuts_ with eye axial length or the days spent in lab (Table 3). However, measurement time and the observer who analyzed the spectra significantly affected the λ_cut_ of the Y-type oil droplets (Table 3). More specifically, the Y-type oil droplet (MWS cone) λ_cut_ was significantly affected by the measurement time, such that the oil droplets measured later had higher λ_cuts_ (β=0.36±0.13) (Table 3).

**Table 3.**
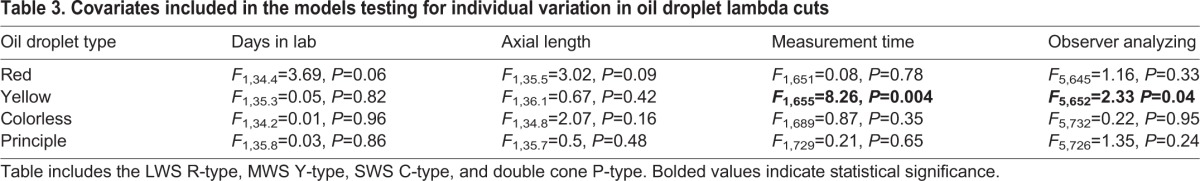
**Covariates included in the models testing for individual variation in oil droplet lambda cuts**

### Visual perceptual modeling

**Fig. 4.**
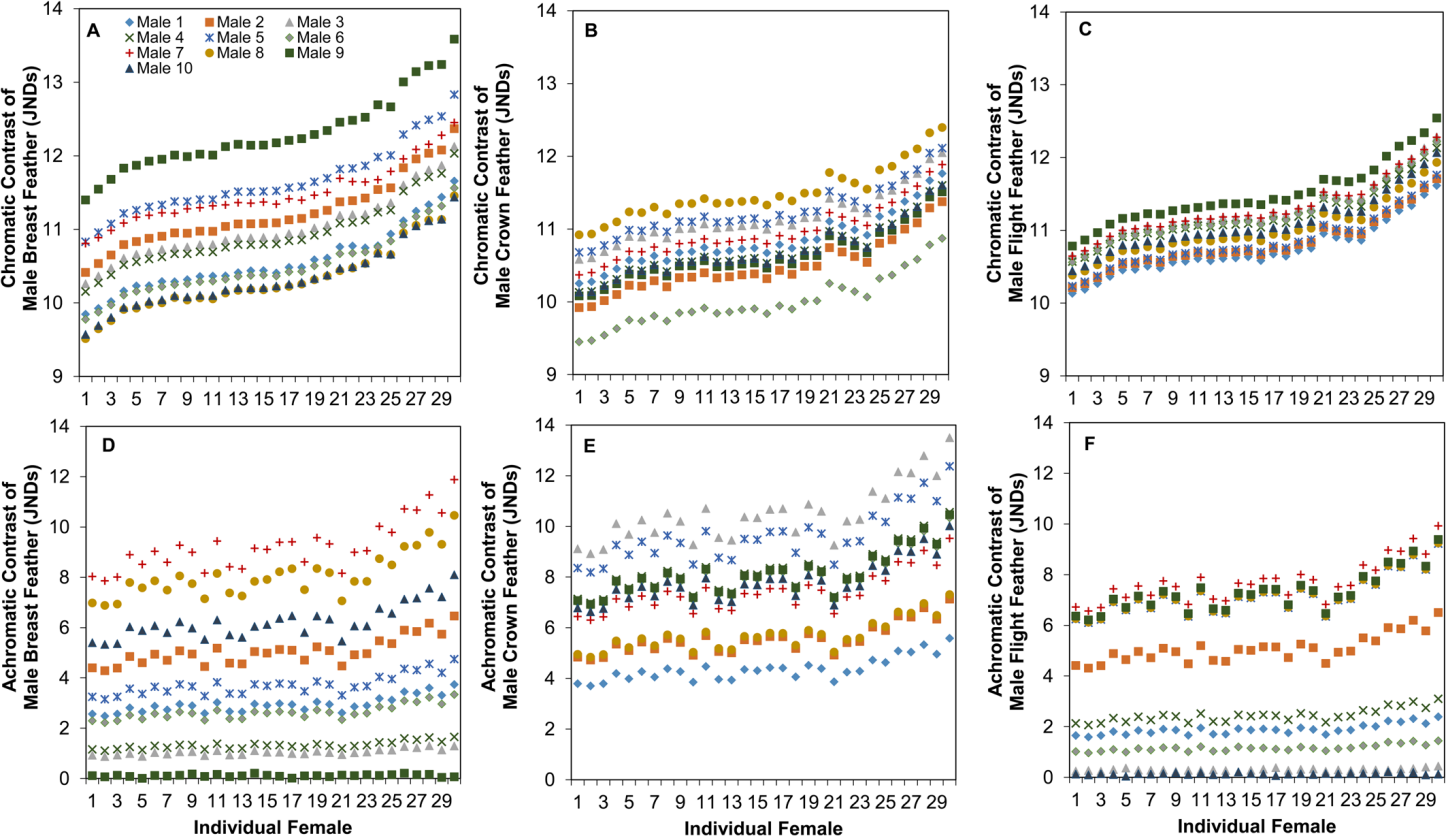
**Variation in female perception of chromatic and achromatic contrasts of male feathers on a grassy background in a shaded patch.** (A-C) Chromatic, (D-F) achromatic. *N*=30. Females varied in their perception of all male feather types: breast feathers (A and D), crown feathers (B and E) and flight feathers (C and F).

**Fig. 5.**
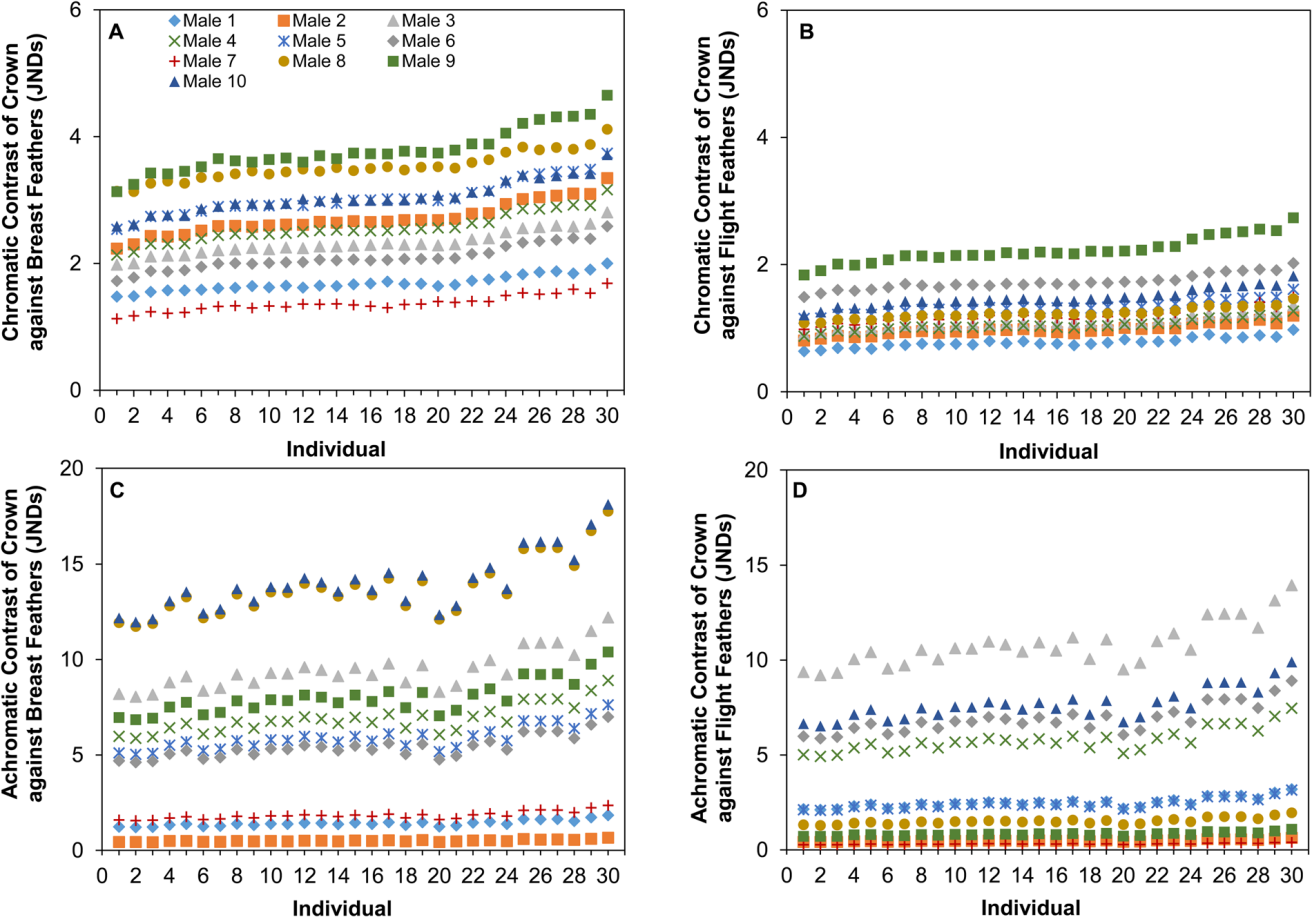
**Variation in female perception of chromatic and achromatic contrasts of male crown feathers against breast and flight feathers.** (A,B) Chromatic, (C,D) achromatic. Contrasts were measured against either the flight feathers (A and C) or breast feathers (B and D). We used the shaded patch ambient light to model perception. Females varied in their perception of male crown patches.

The aforementioned information (cone densities, oil droplet λ_cuts_) of each female allowed us to model their perception of male feather patches against two different backgrounds types: environmental vegetation (e.g. grass or leaves) or male cowbird feather patches (Table 4). For the sake of space, we present in the main text and Fig. 4, female chromatic and achromatic contrast values of male feather patches on a grassy background under shaded light conditions, because results are qualitatively similar across all modeled backgrounds and ambient light conditions (Figs S16-S19). In Fig. 5, we present the chromatic and achromatic contrasts of male crown feathers against both the breast and flight feathers under a shaded patch. Overall, we found that females varied significantly in their chromatic and achromatic perception of all the male feather patches considered (Figs 4 and 5, Table 4; Figs S16-S19). In the analysis of feather patches against a vegetative background, females seem to vary the most in their achromatic perception of male crown feathers against grass compared to the other feather patches (Fig. 4, Table 4). In this scenario, the mean variation in just-noticeable differences (JNDs) across female observers for each male varied by 14% (from 10.00±0.06 to 11.50±0.07 JNDs) in their average chromatic contrasts, and by 40% (from 4.36±0.09 to 10.49±0.21 JNDs) in their average achromatic contrasts. When comparing the contrast of the crown feather patch against flight and wing feathers, females varied more in their chromatic, rather than achromatic, contrast perception (Fig. 5, Table 4). This is despite having relatively low chromatic differences between the feather patches themselves (most males fell below 4 JNDs), indicating that it may be relatively difficult for females to distinguish males based on their chromatic differences. Male identity, background reflectance, and ambient lighting conditions significantly affected female chromatic and achromatic contrast for feather patches against a vegetative patch (Table 5); however, when we modeled chromatic and achromatic contrast of the crown feathers against the flight or breast feathers, we did not find a significant effect of ambient light (Table 5). We found no significant effect of days in the lab and eye axial length across any analyzed conditions (Table 5).

**Table 4.**
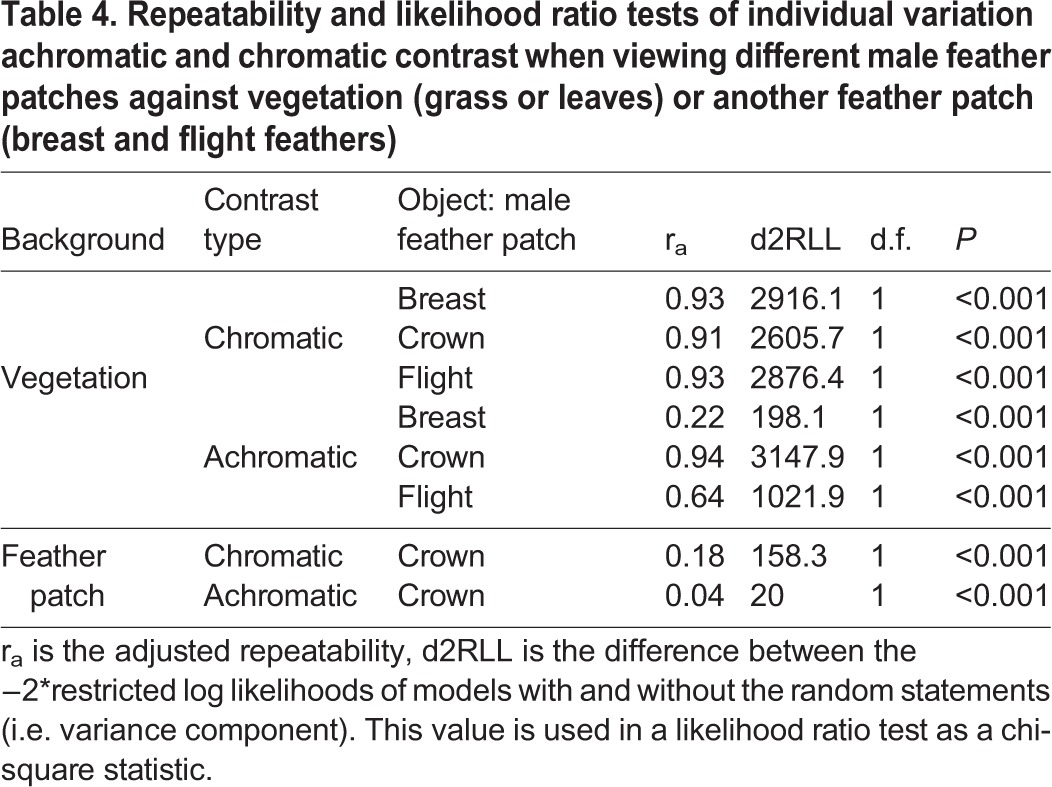
**Repeatability and likelihood ratio tests of individual variation achromatic and chromatic contrast when viewing different male feather patches against vegetation (grass or leaves) or another feather patch (breast and flight feathers)**

**Table 5.**
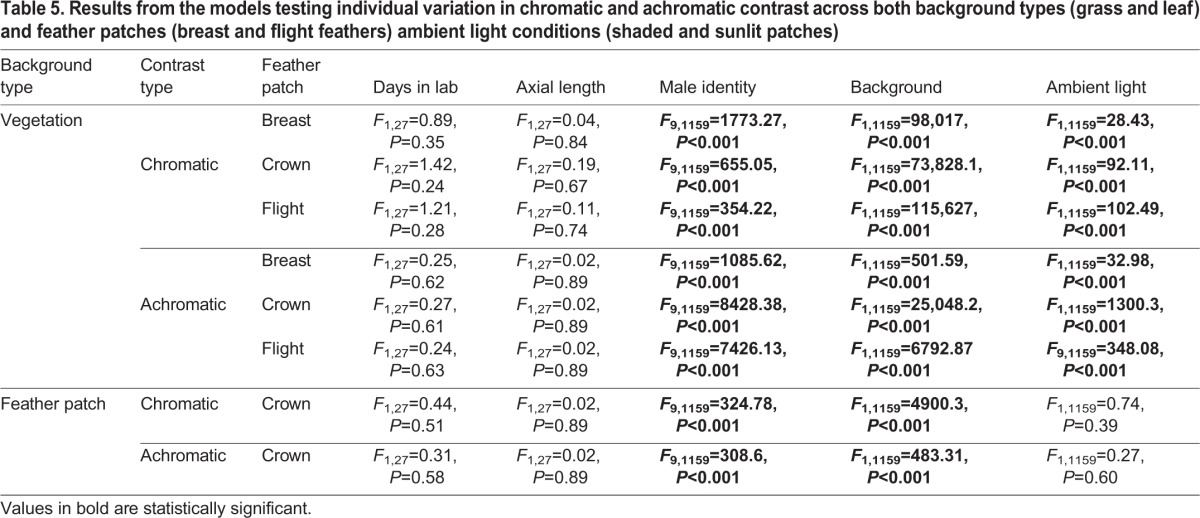
**Results from the models testing individual variation in chromatic and achromatic contrast across both background types (grass and leaf) and feather patches (breast and flight feathers) ambient light conditions (shaded and sunlit patches)**

## DISCUSSION

We found between-individual variation in female cowbirds in two important sensory visual properties associated with visual resolution and chromatic and achromatic visual contrast: cone density (i.e. total density and cone-type proportions) and cone oil droplet lambda cuts. This variation in visual sensory filtering translated to between-individual differences in modeled chromatic and achromatic contrast of male cowbird plumage patches. These findings indicate that the differences in the sensory substrate of receivers may be large enough for females to vary in the way they perceive male visual signals, contradicting one fundamental assumption in animal communication (Ronald et al., 2012).

A few studies have shown data suggesting between-individual variation in visual sensory traits in multiple taxa, including invertebrates (Cotton et al., 2006), fish (Fuller et al., 2004; Parry et al., 2005), birds (Das et al., 1999; Knott et al., 2012; Ensminger and Fernández-Juricic, 2014), and mammals (Jacobs, 1977; Jacobs et al., 1996; Mollon et al., 1984). Nevertheless, few of these studies have explicitly tested whether this variation is statistically significant or if the variation could lead to differences in the perception of signals used in a mate-choice context (but see Ensminger and Fernández-Juricic, 2014). The source of between-individual variation in the sensory system has been linked to many factors including differences in the genetic profile (Jacobs, 1977; Jacobs et al., 1996), condition (Bowmaker et al., 1993; Knott et al., 2010; Toomey and McGraw, 2010), age (Lee et al., 1997; Porciatti et al., 1991), and development (Hart et al., 2006). For example, ambient light availability during development can influence the carotenoid concentration in the cone oil droplets of the domestic chicken (*Gallus gallus domesticus*) (Hart et al., 2006). Although our study was not designed to determine the source of between-individual variation, future studies should explore the fact that cowbird females parasitize nests of multiple other species and therefore cowbird nestlings are raised under very different conditions (e.g. temperature, ambient light properties, access to food, etc.) or that individuals of different ages could have different visual properties.

Our findings on the between-individual variation in the total density and proportions of cowbird cones are similar to those of Ensiminger and Fernández-Juricic (2014) in house sparrows (*Passer domesticus*). However, the range of variation differed between these two songbirds; the adjusted repeatability across cone densities within house sparrows ranged from 0.20 to 0.38 (Ensminger and Fernández-Juricic, 2014), but in the cowbirds ranged from 0.10 to 0.18. Furthermore, individual house sparrows were more repeatable in their cone type proportions, with their adjusted repeatability ranging from 0.17 to 0.47 (Ensminger and Fernández-Juricic, 2014) while cowbirds ranged from 0.05 to 0.2. Both of these values indicate higher levels of between-individual differences in house sparrows compared to brown-headed cowbirds. Interestingly, the greatest amount of between-individual variation in cone density was in the LWS cone type in both cowbirds and house sparrows (Ensminger and Fernández-Juricic, 2014). The variation in cowbird cone density we found could lead to differences between females in their ability to discriminate chromatic and achromatic signals by varying the noise in each cone channel. Receptor noise is equal to: v/√(relative density of a cone type) (Vorobyev and Osorio, 1998). As stated previously, our v parameter was set to 0.15 so we could estimate the range of variation between the maximum and minimum relative densities from each cone type. Based on our data, receptor noise could vary in the LWS cone receptor channel by up to 36%, by 33% in the MWS cone receptor channel, by 27% in the SWS cone channel, and 33% in the double cone channel (based on Vorobyev and Osorio, 1998). Unfortunately, studies assessing the effects of noise variation in each photoreceptor channel on a behavioral response are quite limited (Olsson et al., 2015).

Additionally, the variation in cone density could affect the ability of different females to resolve variations in male visual signals. For instance, following Tamura and Wisby (1963), we estimated spatial resolving power in female cowbirds to vary between 9.75 to 13.5 cycles per degree. If we consider the average separation of feather barbs during puffing to be around 1 mm, females with the lowest and highest spatial resolving power would need to be at least 55 and 78 cm away from the displaying male to resolve this level of detail, respectively. As males typically display to females from less than one meter (Rothstein et al., 1988), this difference in spatial resolving power may challenge the visual system of some females to assess males.

We also found significant between-individual variation in the λ_cut_ properties of every cone oil droplet we tested in the central region of the retina. Previous work has noted that both diet (Bowmaker et al., 1993; Knott et al., 2012) and light exposure (Hart et al., 2006; but see Toomey and McGraw, 2016) can alter retinal carotenoid levels, but these studies did not examine individual variation. Nevertheless, all the birds in this study were fed the same diet and exposed to the same lighting regimes, suggesting that there may be additional mechanisms by which individual variation in oil droplet absorbance can occur. Oil droplet absorbance can also vary by retinal region (Hart, 2004; Knott et al., 2012); here we focused on the central region of the retina containing the fovea, but it is likely cowbird oil droplet absorbance also varies according to location. Our statistical test, however, compares the level of within-individual variation to between-individual variation; as we found statistically significant results, this indicate that the between-individual differences are greater than any within-individual differences we measured.

We found that the greatest amount of between-individual variation in λ_cut_ was in the Y-type oil droplet (adjusted repeatability=0.48). Interestingly, past studies have also shown larger standard deviations in the Y-type oil droplet relative to other single cone oil droplets, although this parameter does not indicate whether this variation was significant (Hart et al., 1998; 2000a,b; Hart, 2004; Baumhardt et al., 2014). The Y-type oil droplet is specifically associated with the MWS cone that is most sensitive around 506 nm (i.e. green in the visible light spectrum, and the color of the iridescent sheen in male cowbirds). As the hue of this iridescent coloration has been linked to male cowbird condition and may serve as an honest signal during mate choice (McGraw et al., 2002), variation in this oil droplet may have particularly important implications for how female brown-headed cowbirds view potential mates.

The results reported above were found after controlling statistically for the confounding effects of different factors. Eccentricity effects reflected the decrease in cone density from the fovea to the periphery of the retina (Walls, 1942). By including this factor in the statistical model we were in essence removing any between-individual variation that was due to eccentricity. Additionally, observer significantly affected the counts, as found in a previous study (Ensminger and Fernández-Juricic, 2014); however, including this fixed factor allowed us to remove its confounding effects in the model. Each observer did not count a full individual retina; instead, we randomized the sites, retinas, and individuals assigned to each person. Consequently, we have no reason to think that observer effects biased our results on between-individual variation. We also found that microspectrophotometry (MSP) measurement time since retinal extraction affected the Y-type oil droplet absorbance, perhaps as the retina prep started drying during the procedure.

Finally, we showed that between-female variation in both cone density and oil droplet lambda cuts can lead to differences in female visual perception of male signals using established chromatic and achromatic contrast modeling. These individual differences were found when modeling across different lighting conditions and background substrates (vegetative or plumage based). This sets the basis for the possibility that females vary their mating preferences based on the ability of their sensory system to distinguish between subtle differences in signals from different males. A recent study on *Anolis sagrei* lizards found differences in the mean probability of detection based on even relatively small differences in JNDs (e.g. 2–4 JNDs); indicating that even relatively small changes in chromatic contrast values like those we find in our study could translate to behaviorally different outcomes (Fleishman et al., 2016). It is possible that female cowbirds unable to discriminate between males may choose mates randomly, or exert less choosiness and invest less time in sampling different males based on that trait. Furthermore, if males display a multimodal signal, like cowbirds do by pairing a visual display with a song, females unable to discriminate males in one modality may rely more on the other modality to evaluate potential mates (reviewed in Ronald et al., 2012).

Interestingly, our data show that despite individual differences among females, within a given ambient lighting, feather patch, and background combination, a given male shows consistently higher contrast across females. Furthermore, across our different environmental conditions investigated (i.e. grassy or leaf substrate under either a sunlit or shaded patch), this single male remained consistent. This suggests that in our conditions measured, across all females, a single male may be perceived as the most salient. Perhaps if we had included backgrounds that differed more in their reflectance (e.g. more variation in hue), or challenged the visual system by testing very dim lighting conditions, this finding may be different. Very few studies have addressed the question of how a female's sensory biology interacts with environmental context to influence mate choice. There is, however, evidence that individual preference strength is positively correlated with female eye span, and therefore perhaps visual acuity, in the stalk-eyed fly (*Diasemopsis meigenii*) (Cotton et al., 2006). Moreover, female house finches (*Carpodacus mexicanus*) given a low-carotenoid diet had lower retinal carotenoid levels (Toomey and McGraw, 2010) and showed lower mating responsiveness (Toomey and McGraw, 2012). It is important to note here that our data make no suggestion that male contrast is indicative of their condition or quality as a mate. Rather our data only suggest that females may perceive males differently, which, combined with the variety of ambient light and environmental backgrounds animals face in the wild, may influence their mate-choice decisions.

In conclusion, we have demonstrated that the assumption that all females process male signals equally does not appear to hold in cowbirds. This can ultimately have multiple implications for the evolution of male signals in this species; for example, male visual signals may not be under strict directional selection as individual females may rank male signals differently. Furthermore, recent work has shown that female mate preferences in this species is influenced by an interaction between male visual and acoustic signals (Ronald et al., 2017). Ultimately, differences between females in visual perception may be exaggerated by this interaction between multimodal signal components. One implication is that males may adopt different signaling strategies to reach different female receivers. This is a promising avenue for future research that would shed light on the evolution of mating signals.

## MATERIALS AND METHODS

### Animal capture and housing

All animal care and experimental procedures were approved by Purdue University Animal Care and Use Committee (PACUC) Protocol # 1111000151. Ten male cowbirds were wild-caught in decoy traps by the USDA APHIS (Sandusky, OH, USA) in May 2011 to measure male feather reflectance. Thirty-eight adult (>2 years of age) female cowbirds were caught in the same location in May 2013 to measure cone densities and oil droplet absorbance. All birds were banded and housed at Purdue University in individual enclosures (64 cm×40 cm×64 cm). All birds were exposed to the same feeding and light-level regimes. Birds were provided *ad libitum* access to mixed seed, grit, and water. The lighting schedule followed the natural lighting conditions of West Lafayette, IN, USA (i.e. schedule was adjusted weekly and ranged from 14 h light:10 h dark in the summer to 10 h light:14 h dark during the winter). Females were used in a mate-choice experiment described elsewhere (Ronald et al., 2017) prior to visual traits characterization.

### Cone densities and proportions

Thirty left-eye retinas were used to measure cone densities and their proportions (see Fig. S1 for a schematic representation of the retinal regions sampled). We used only left eyes for cone density and proportion estimates to minimize the possibility that interocular variability would inflate our estimates of between-individual variation. Furthermore, we needed to use the other eye to model individual variation in oil droplet absorbance. We thus assumed that any laterality between individuals was consistent across individuals. We followed standard procedures described thoroughly elsewhere for retinal extraction (Ullmann et al., 2012) and cone density estimation (Ensminger and Fernández-Juricic, 2014). We briefly summarize them here. Following euthanasia via CO_2_ asphyxiation, we measured eye axial length (in mm) with digital calipers (0.01 mm accuracy) and then stored the right eye in phosphate buffered saline solution (PBS) in the refrigerator for approximately 4 h while the left eye was processed. We then hemisected the left eye with a razor blade just posterior to the lens at the *ora serrata*. The vitreous humour was removed with tweezers and spring scissors under a dissecting scope and then the eyecup was saturated with PBS. The retina was removed without touching the retinal tissue directly as oil droplets can easily detach from the retina (Hart, 2001b; Kram et al., 2010). We first detached the choroid from the sclera and then severed the optic nerve. We removed the retinal tissue by holding on to the optic nerve and separating the tissue from the eye cup (Ullmann et al., 2012). During this step, the pigmented epithelium (i.e. layer that nourishes the retina) often spontaneously detached from the retinal tissue. After removal from the eyecup, we attempted to remove any remaining pigmented epithelium from the retina with two sets of tweezers, pulling the epithelium in opposite directions without touching the retinal tissue (Ullmann et al., 2012). Any pigmented epithelium that remained after this was not removed. Using two paintbrushes, we then carefully transferred the retina to 4% paraformaldehyde for 30 min to strengthen the tissue and preserve the retinal matrix (Ensminger and Fernández-Juricic, 2014; Hart, 2001a). Afterwards, we flattened the retina (vitread-side up) on a slide by making small radial incisions and gently unrolling the retinal edges with a fine-tipped paintbrush. We added two drops of PBS, placed a coverslip on top of the retina and then gently flipped the coverslip over with the retina attached to it. More PBS would have made it difficult to focus on retinal tissue so we were conservative in our application. The coverslip was then adhered to the slide with superglue so that the retina was sclerad-side up. We then added four drops of superglue at the corners and gently placed an additional coverslip on top of the retina; we allowed the super glue to dry to help prevent the retina from being compressed between the two coverslips. Moreover, none of our photos showed evidence of oil droplets bursting, which can result when too much pressure is placed on the retinal tissue.

Retinal photographs were completed no later than 2.5 h after preparation; this strict time limit was needed to prevent the retina from becoming desiccated which can affect the visualization of the oil droplets (Ullmann et al., 2012; Ensminger and Fernández-Juricic, 2014). Retinas were viewed with an Olympus BX51 microscope and the SRS (Systematic Random Sampling) Image Series Acquire workflow of Stereo Investigator v.10 (MBF Bioscience, Williston, VT, USA). This workflow allowed us to first trace the perimeter of the retina and eliminate any areas that were obstructed with pigmented epithelium that was not removed during the extraction procedure described above. We then fit a systematic random grid (250 squares) over this traced retina; the average square size per retina was 0.45±0.006 mm^2^. We used a continuous sampling grid across the entire retinal area to avoid introducing bias, or over-sampling of particular regions, to our counting efforts. We set the following stereological parameters: area sampling fraction (asf; the ratio of the counting frame area to the grid area)=0.005±6.9×10^−5^ per retina, number of sections=1, stereological sampling fraction=1 per retina, thickness=1, and thickness of sampling fraction=1 per retina (West, 2013; Bonthius et al., 2004). Pictures at a retinal site were taken using a 40× objective lens with a numerical aperture of 0.1 under both brightfield and epiflourescent illumination. The counting frame (50 μm×50 μm; 0.0025 mm^2^) was always located in the upper left corner of all the sites.

Following a previous study (Ensminger and Fernández-Juricic, 2014), we concentrated our cone counts on the central region of the retina, also known as the perifoveal region. This portion of the cowbird retina holds the fovea which is located inside a larger area centralis (Dolan and Fernández-Juricic, 2010; Fernández-Juricic et al., 2013). The fovea is the center of high visual resolution because of the high density of cone and retinal ganglion cells (Collin, 1999). This is the most important region of the retina to investigate between-individual variations because the avian fovea has been suggested to be (1) the center of chromatic and achromatic vision (Fernández-Juricic et al., 2013; Baumhardt et al., 2014), and (2) the center of visual attention (Tyrrell et al., 2014, 2015). Consequently, we assumed that females in a mate-choice task would use their center of acute vision to assess males visually. This assumption has some empirical support (Yorzinski et al., 2013). Furthermore, past studies in humans have shown that the highest degree of between-individual variation in cone density occurs in the perifoveal region (Curcio et al., 1990).

Fovea location was determined for each retina using the tip of the pecten and its angle as landmarks (Ensminger and Fernández-Juricic, 2014) (see Fig. S1). To determine fovea location we used images from whole-mounted and cresyl-violet stained retinas as described by Ullmann et al. (2012). This technique stains retinal ganglion cells which are absent from the fovea, allowing us to identify the fovea as an unstained region and the pecten. We found that the cowbird fovea is on average 1840±5.3 μm from the pecten tip, at 103±0.43° angle. We used these values to estimate the fovea location for all of our individuals used in this study. We chose to count cells in sites that lay within a 2500 μm radius from the fovea (i.e. within the perifoveal region) which is approximately 12% of the total area of the retina (Fig. S1). We chose this size radius for two reasons: (1) we wanted to be fairly consistent with previous studies that used a 1600 μm radius in house sparrows, which was approximately 9% of the total retinal area, and (2) we wanted an adequate number of sampling regions from within this area (each individual had an average of 29.6±1.6 sites counted) (Fig. S1).

Cone densities and proportions were estimated by counting the different cone oil droplets, which are organelles located in the distal end of the inner segment of all cone types. Each oil droplet type contains different types and concentrations of carotenoids (Hart, 2001b; Bowmaker et al., 1997). In birds, each oil droplet is associated with a specific type of cones, which allowed us to estimate cone density from oil droplet counts (see Fig. S2 and Table S1 for representative images). Birds are tetrachromats and therefore have four single cones used in color vision: the ultra-violet sensitive (UVS) cone with a transparent oil droplet (i.e. T-type oil droplet), the short-wavelength sensitive (SWS) cone with a colorless oil droplet (i.e. C-type oil droplet), the medium-wavelength (MWS) sensitive cone with a yellow oil droplet (i.e. Y-type oil droplet), and the long-wavelength sensitive (LWS) cone with a red oil droplet (i.e. R-type oil droplet). Finally, birds also have a double cone, thought to aid in achromatic vision and motion detection (Vorobyev et al., 1998), and the principal member of the double cone is associated with a principle oil droplet (i.e. P-type oil droplet). Following the parameters established in Hart (2001a), we identified oil droplets based on color, size, and plane of the retina as we have done in previous studies (Moore et al., 2012; Ensminger and Fernández-Juricic, 2014; Baumhardt et al., 2014). ImageJ (http://rsbweb.nih.gov/ij/) was used to count oil droplets within each site; these values were then used to calculate the density of cones at each sampled site (number of cells counted/mm^2^). The proportions of each cone type were calculated at each site by dividing the cone density of a given cone type by the total density of all cone types. A total of seven different observers were trained on 83 different training sites and all observers had counting repeatabilities of >0.9 compared to K.L.R. Sites were randomized prior to being analyzed by an observer such that no one individual analyzed a single bird.

A total of 1163 sites from 30 individual retinas were included in our initial sampling. To eliminate sites where the oil droplets may have detached from the retina and thus bias our results (Hart, 2001b; Kram et al., 2010), we refined our sampling efforts to only include sites that followed these strict criteria. We only included sites where (1) each cone type was present, (2) cones were arranged in a matrix-like pattern (e.g. Kram et al., 2010), and (3) no pigmented epithelium obstructed the counting frame. Following these criteria, we eliminated 258 sites. If any part of the site did not meet these requirements, we divided the counting frame into four quadrants and only counted the quadrants that met those criteria. In total, we counted an average of 5264±383 cells per individual (ΣQ-), with sites containing an average of 170.3±6.7 cells and an observed coefficient of variation of group mean (CV)=0.41±0.02. We calculated two parameters of stereological reliability of our estimates: first, the Sheaffer-Mendenhall-Ott coefficient of error (CE) was 0.08±0.006; values <0.1 are considered highly reliable (Glaser and Wilson, 1998). Second, we calculated the Sheaffer-Mendengall-Ott CE2/CV2 (i.e. an estimate of the amount of variation in cell counts due to sampling errors caused by stereological procedures) to be 0.04±0.002. Here, values of <0.5 are considered highly reliable (Glaser and Wilson, 1998). Together, these parameters indicate our estimates are highly reliable and that our sampling efforts were adequate.

### Oil droplet absorbance

Thirty-eight right-eye retinas were used for measuring oil droplet absorbance using microspectrophotometry (MSP) following Fernández-Juricic et al. (2013). The eye was hemisected, and the retina removed and flattened, following the same procedures as described above (Ullmann et al., 2012). We removed two retinal pieces (later referred to as preps 1 and 2) of approximately 3 mm^2^ from the center of the eye (see Fig. S1 for a schematic representation). We placed these preps onto separate Corning No. 1 22×33 glass slides where they were macerated with a razor blade. We covered each prep with a cover slip and sealed it with black nail lacquer to prevent desiccation.

We took MSP measurements of the oil droplet absorbance (Liebman, 1972) under dim red-light from a custom-made microspectrophotometer (Dr Ellis Loew, Cornell University, Ithaca, NY, USA; design described in McFarland and Loew, 1994). Oil droplets were viewed with a Zeiss Ultrafluar Glyc objective (32×, NA 0.4) as the condenser with a drop of glycerol added to the condenser, and a dry objective (80×, NA 0.9). Images were projected on an 8″ TFT Color LCD Monitor via an EXVision Super Circuits CCD camera. Upon identifying an oil droplet, a baseline measurement in an empty portion of the prep was recorded. We then took a measurement of the oil droplet absorbance in 1 nm increments from 350-750 nm. Based on the shape of the absorbance curve during collection, we could distinguish R- and Y-type oil droplets, but P- and C-types were more difficult to distinguish from each other (see Figs S3-S14 for representative MSP spectra). To address this, we ran a cluster analysis (described below) based on several other parameters to differentiate statistically between C- and P-type oil droplets.

We collected 40 oil droplet absorbance spectra from the prep 1 (i.e. approximately 10 examples of each oil droplet: R-, Y-, C-, and P-types) before repeating this with prep 2. We did not attempt to collect T-type oil droplets because they do not absorb light above 350 nm due to the lack of carotenoids in the oil droplet (Hart, 2001a). This means that the absorbance spectra of the T-type does not contain a peak. After taking the normalized absorbance measurements we used a spectra analysis program, OilDropSpec (Fernández-Juricic et al., 2013) to characterize each oil droplet using several established parameters: λ_cut_, the wavelength where 100% of the light is absorbed by the oil droplet; λ_mid_, the wavelength where 50% of the light is absorbed; and λ_0_, the wavelength where transmittance equals 1/e (Lipetz, 1984; Hart and Vorobyev, 2005; Fernández-Juricic et al., 2013). The observer first sets a baseline of the absorbance spectra and then the program normalizes the long wavelength arm of a spectrum to one and determines the wavelength at which the absorbance is 50% (λ_mid_) between the baseline and peak absorbance of the curve. The observer is then able to approve or modify the peak the program has specified; once the peak is chosen the program fits a trend line to the absorbance data 10 nm on either side of λ_mid_, and records the intercept, slope, and R^2^ parameters of the trend line. We eliminated oil droplets from further analysis if this R^2^ value was below 0.85 (i.e. when the spectra included a lot of scatter). Using these trend-line parameters, the program determines the wavelength at which the absorbance=1 (i.e. λ_cut_) and calculates *B*_mid,_ the slope of the transmittance at λ_mid_ (Hart and Vorobyev, 2005). A total of six different observers were trained on how to use the OilDropSpec program on 180 unique oil droplets. Before analyses began all observers had repeatabilities of >0.9 compared to K.L.R. Oil droplet spectra were randomized prior to being analyzed by an observer such that no one individual analyzed a single bird.

As noted above, P- and C-type oil droplets were difficult to distinguish from one another because their range of λ_mid_ and λ_cut_ values tend to overlap (Hart and Vorobyev, 2005). To differentiate between these oil droplet types, we first ran a principle components analysis (PCA) for each individual separately with Proc PRINCOMP in SAS v. 9.3 (SAS Institute Inc., Cary, NC, USA) on each λ_cut_ value (in 1 nm increments) from 350 nm to 475 nm for any spectra that was either a P- or C-type. On average, the eigenvalue for factor 1 from these PCAs was 47.65±1.19 and explained 38.12±0.01% of the variation. Higher values of PCA factor 1 were positively correlated with higher λ_mid_ values. We then used this PCA factor 1 value, the *B*_mid_, and λ_mid_ for each oil droplet to run a cluster analysis (Proc FASTCLUS in SAS) and sort the oil droplets into two clusters and statistically distinguish between C- and P-type oil droplets. We decided to use λ_mid_ and *B*_mid_ instead of λ_cut_ because λ_mid_ is calculated independently within the OilDropSpec (Fernández-Juricic et al., 2013) whereas λ_cut_ is derived from both λ_mid_ and B_mid_. Each oil droplet was assigned into one of two clusters (i.e. the C-type cluster or P-type cluster) using the nearest centroid sorting (Fernández-Juricic et al., 2013). After the cluster analysis, we included in our analyses of between-individual variation 748 C-types (19±1 per bird), 680 Y-types (17±1 per bird), 670 R-types (17±1 per bird), and 769 P-Types (20±1 per bird).

### Perceptual modeling

We used perceptual modeling to examine whether individual differences in cone density and oil droplet λ_cuts_ can contribute to individual differences in visual perception. To do this we used a tetrachromatic avian visual model where chromatic and achromatic contrast (i.e. the distance in avian color space between an object and the background) is limited by photoreceptor and neural noise and is a function of (1) object reflectance, (2) background reflectance, and (3) ambient light illumination (Vorobyev and Osorio, 1998). This modeling approach has been extensively used in mate-choice research to understand female perception of male signals across different conditions (e.g. Morehouse and Rutowski, 2010; Sztatecsny et al., 2010; Uy and Endler, 2004; Vorobyev and Osorio, 1998; Vorobyev et al., 1998). Moreover, it is the only modeling approach that allows the user to input the spectral absorbance curves of photoreceptors, oil droplet absorbance values, and relative photoreceptor densities. A recent study found that this model was found to be accurate in terms of predicting behavioral results and produced results similar to another visual model commonly used (i.e. the tetrahedral color space model) (Fleishman et al., 2016).

We estimated chromatic and achromatic contrasts of different male patches from the visual perspective of different individual females (i.e. considering the visual traits we measured for each female). More specifically, we modeled (1) how male breast, crown, and flight feathers contrasted against two vegetation backgrounds (grass and leaves), and (2) how male crown feathers contrasted against male breast and flight feathers. We modeled both of these scenarios under two ambient light conditions (sunlit patch and shaded patch). We chose these conditions because male cowbirds are known to display to females both while on the ground and while perching in trees (Rothstein et al., 1988), and to compare color patches within an individual following previous methodology (Endler, 1990) (see Fig. S15 for the reflectance spectra of each feather patch and measured background reflectance). In the Results, we present figures representing the chromatic and achromatic contrast values calculated under a shaded-patch for (1) feathers (i.e. crown, flight, and breast) against a grassy background, and (2) crown feathers against breast and flight feathers. All the other conditions of a male against vegetation (grass background and sunlit patch, leaf background and sunlit patch, leaf background and shaded patch) and male crown feathers against breast and flight feathers in a sunlit patch also show that visual perception varied significantly between individuals (see Figs S16-S19).

Male breast, crown, and flight feathers are likely to be used as signals in both female mate-choice and male-male aggressive interactions (Rothstein et al., 1988). Feathers from each of these patches were collected in mid-November 2011 from 10 adult males that had finished molting. All feathers were stored individually in non-acidic envelopes at room temperature until reflectance measurements were taken two weeks later with a custom-made goniometer (design described in Meadows et al., 2011). The goniometer was attached to an Avantes spectrophotometer and PX-2 pulsed xenon light source (Avantes, Louisville, CO, USA). Single feathers were mounted on a black velvet background via the non-iridescent proximal end of the feather to avoid damaging the color-producing microstructures. Mounted feathers were placed on the specimen stage and tilted (at an angle of 4.1±0.7°) until the maximum reflectance peak was reached (Meadows et al., 2011). This measurement procedure allowed us to record the reflectance of the iridescent cowbird feathers in a standardized and repeatable way (Meadows et al., 2011). Measurements were recorded in a dark room and taken relative to a magnesium oxide white standard (Avantes WS-2). All reflectance measurement data (percent reflectance) were collected from 300-700 nm, within the avian visual range (Cuthill, 2006; Mullen and Pohland, 2008), and reflectance was binned in 1-nm increments.

Vegetation background reflectance measurements were taken with an Ocean Optics Jaz spectrometer (Ocean Optics Inc. Dunedin, FL, USA). The bifurcated reflectance probe using a combination of Tungsten and Deuterium light source was held at a 45° angle to the background substrate and 20 reflectance measurements were taken from 300-700 nm in 1-nm increments. A dark reference and white reference (∼97% reflected Halon standard) were collected to calculate percent reflectance. The 20 measurements were subsequently averaged.

Ambient light irradiance measurements were collected with a StellarNet Black Comet spectrometer (StellarNet Inc. Tampa, FL, USA) with an irradiance probe with a cosine corrector under a tree canopy (i.e. shaded patch) and in the middle of a soybean field (i.e. sunlit patch) on a bright, sunny day. Light was measured twice in each of the four cardinal directions parallel to the ground and then directly up at the sky. All measurements were averaged to give irradiance data from 300-700 nm.

We estimated chromatic and achromatic contrasts using the ‘pavo’ package in R (Maia et al., 2013 and R Development Core Team). All models were calculated in terms of relative quantum catches following Vorobyev et al. (1998). This model considers the following physiological parameters: (1) the sensitivity of the cone visual pigments (i.e. λ_max_), (2) the absorbance properties of the SWS, MWS, LWS, and principle oil droplets (i.e. λ_cut_ and *B*_mid_), (3) the densities of the cones, and (4) the transmittance of the ocular media (Govardovskii et al., 2000; Hart and Vorobyev, 2005; Maia et al., 2013). We modeled each individual female receiver separately, using their specific values of λ_cut_, *B*_mid_, and relative cone densities (Table S2). We used published data on the brown-headed cowbird single-cone sensitivities: 369 nm (UVS), 475 nm (SWS) 506 nm (MWS), and 573 nm (LWS) (Fernández-Juricic et al., 2013). Double-cone sensitivity was assumed to be similar to the LWS visual pigment (following Hart, 2001a) as we did not have λ_max_ values for the double cones (Fernández-Juricic et al., 2013).

Moreover, we used ocular media transmittance measured from three eyes across two individual cowbirds not included in this study following the procedures described in Hart (2004) and Håstad et al. (2009). Transmittance was recorded with a StellarNet black comet spectrometer (StellarNet Inc. Tampa, FL, USA) using a combination Tungsten and Deuterium light source from 300-700 nm. Cowbird single-cone sensitivities and ocular media transmittance were kept constant across all females modeled, as they have been shown to vary little within species (Hart et al., 2006).

Pavo uses three ordered functions: (1) The sensmodel, which models spectral sensitivity based on the four peak cone sensitivities from the combination of visual pigment and oil droplet absorbances, *B*_mid_ values, and the ocular media transmittance (Govardovskii et al., 2000; Hart and Vorobyev, 2005). (2) The vismodel, which uses the output of the sensmodel and the reflectance of the object, background, and the ambient light irradiance to calculate quantum catches for each of the four single cones across the avian visual spectrum (300-700 nm) (see Eqn 1 from Vorobyev et al., 1998). Within the vismodel function, Pavo uses a log transformation of the quantum catches to make the differences in cone stimulation proportional to their magnitudes (Weber-Fechner law, see Eqn 4 in Vorobyev et al., 1998). (3) The coldist function, which uses the output of the vismodel, the relative cone densities, and the Weber fraction to calculate chromatic or achromatic contrast. In this coldist function we specified that the model consider the total attenuation of light intensity resulting from having a pass filter by indicating that noise=‘neural’, which assumes that under the ambient light condition that the receptors would be saturated (this assumption is very realistic except under very dim lighting conditions which we did not test). We used data from the four single cones (UVS, SWS, MWS, and LWS) to calculate chromatic contrast, and data from the double cones to calculate achromatic contrast following Siddiqi et al. (2004).

To calculate chromatic contrast for each female, we included our measured values of oil droplet absorbance (λ_cut_), *B*_mid,_ and relative cone densities for the UVS, SWS, MWS, and LWS cones (Table S2). Relative cone densities were calculated by dividing each absolute cone density value by the UVS cone density. Thus, relative values were always equal to 1 for the UVS cone type, and higher values for the other cone types. To calculate achromatic contrast with user-defined input for the double cones, we included data from the SWS (475 nm), MWS, (506 nm), LWS (573 nm), and double cones (573 nm), and the oil droplet λ_cuts_, *B*_mid_, and relative densities of these cones for each individual (Table S2). Additionally, we set our v argument (associated with the Weber fraction) to 0.15, calculated from the equation v=0.1(/√(LWS:UVS cone ratio) (Maia et al., 2013). For avian models, Pavo assumes the standard deviation of the noise to be 0.1; this follows the estimate of the Weber fraction calculated from a bird species (*Leiothrix lutea*) (Vorobyev et al., 1998). In our calculations we used the average LWS to UVS cone ratio, which was (2.34±0.09).

### Statistical analyses

To test for between-individual variation in cone densities and proportions, oil droplet λ_cuts_, and chromatic and achromatic contrasts, we used general linear mixed models in SAS version 9.4 (SAS Institute Inc., Cary, NC, USA). We tested for significant between-individual variation with Likelihood Ratio tests by comparing the –2*restricted log likelihood parameter between models with and without the random effect (i.e. ‘bird identity’) (West et al., 2007). We used the difference between these values from the two models as a Chi square statistic in the Likelihood Ratio test, with d.f.=1; a significant Chi square indicates significant between-individual variation. We also calculated the adjusted repeatability to estimate the degree to which individuals differed from one another (Nakagawa and Schielzeth, 2010) by dividing the between-individual variance estimate by the sum of that variance and the residual variance estimates in the full model (Nakagawa and Schielzeth, 2010; Ensminger and Fernández-Juricic, 2014). The fixed effects included were different depending on which response variable was being modeled, so we discuss those separately below for each response variable. In all models, we specified the Kenward-Roger method to calculate the degrees of freedom for the fixed effects.

In addition to examining total cone densities (all photoreceptors grouped together), we assessed each type of cone separately. Cone type proportions were calculated by dividing the number of cones of a specific type, at specific site, by the total number of all cones (the sum of single and double cones) at that site. We also included several fixed effects as covariates in the models: (1) eccentricity (i.e. the distance from the fovea to the retinal site) because cone density changes across the retina (Walls, 1942), (2) the observer (*N*=7) who counted a given site as Ensminger and Fernández-Juricic (2014) found significant between-observer differences, (3) the number of days the bird was in the lab because in our experience as the length of time in lab increases it also becomes more difficult to remove the pigmented epithelium (i.e. the pigmented cell layer that nourishes the retina and is firmly attached to the choroid) from the retina and we wanted to control for this factor (Ensminger and Fernández-Juricic, 2014), and (4) eye axial length as a proxy for eye size, because larger eyes can have a higher overall number of cones (Tamura and Wisby, 1963).

Individual variation in oil droplet λ_cut_ was calculated separately for each oil droplet type. We decided to investigate λ_cut_ because at this wavelength no more light is transferred to the visual pigments, and consequently it is the most commonly used metric to describe oil droplet absorbance (Hart et al., 1999). In these analyses, the number of days the bird was in the lab was included as a covariate because oil droplet λ_cuts_ may change with time spent in the laboratory (Toomey and McGraw, 2016). Additionally, we also included individual eye axial length, the time of the day oil droplet λ_cut_ was collected (to control for any changes in lambda cut as the prep dries over time), and the observer who used the OilDropSpec Program to analyze each oil droplet spectra (*N*=6 observers).

We obtained 300 different estimates of chromatic and achromatic contrasts: 30 different females×10 different males. We calculated individual variation separately for each feather patch type we analyzed. We included the following fixed effects: background type (i.e. grass, leaf, flight feather, breast feather), ambient light condition (sunlit patch, shaded patch), days an individual female spent in the lab, female eye axial length, and male identity because we expected different males to have different feather reflectance properties. All results include mean±s.e.m. To examine the direction of the relationship of any continuous fixed effect we examined β, the slope of the predicted line describing the relationship between the continuous independent and the dependent factors.

## Supplementary Material

Supplementary information
