# Supplementary material for: Testing a key assumption in animal communication: between-individual variation in female visual systems alters perception of male signals
Source: Biol Open. 2017 Dec 15;6(12):1771–83. doi: 10.1242/bio.028282 (PMC5769651; doi:10.1242/bio.028282)
Supplement: Supplementary information [file biolopen-6-028282-s1.pdf]

Figure S1

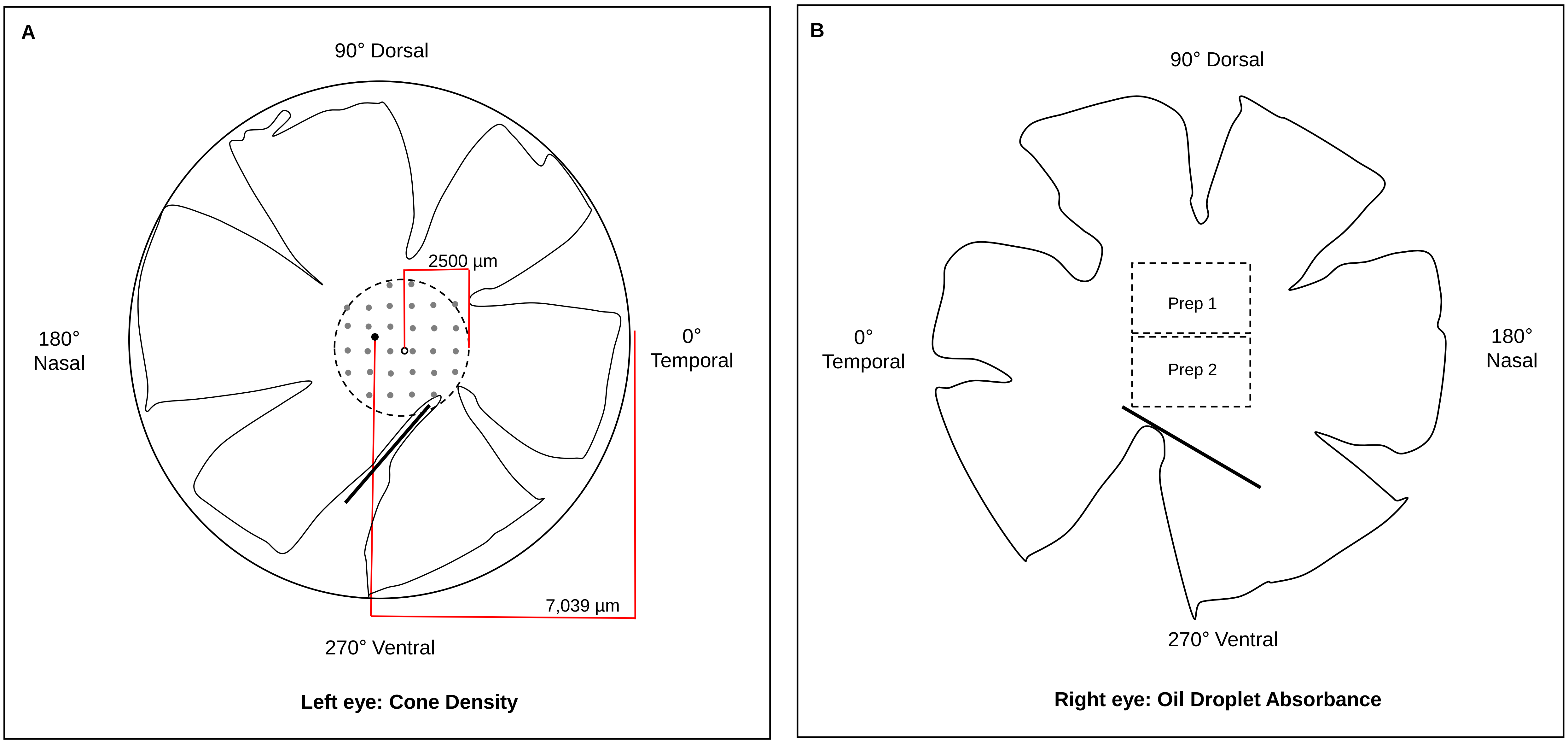

Figure S1. Schematic example of a retina from the left eye (A) to be used for cone density determination and the right eye (B) to be used for oil droplet absorbance determination. In A, the open circle represents the fovea while the closed circle represents center of the retina. The dotted line indicates the sampling area of 2500 um diameter around the fovea. In both illustrations the pectin is represented by a thick, black bar.

Figure S2

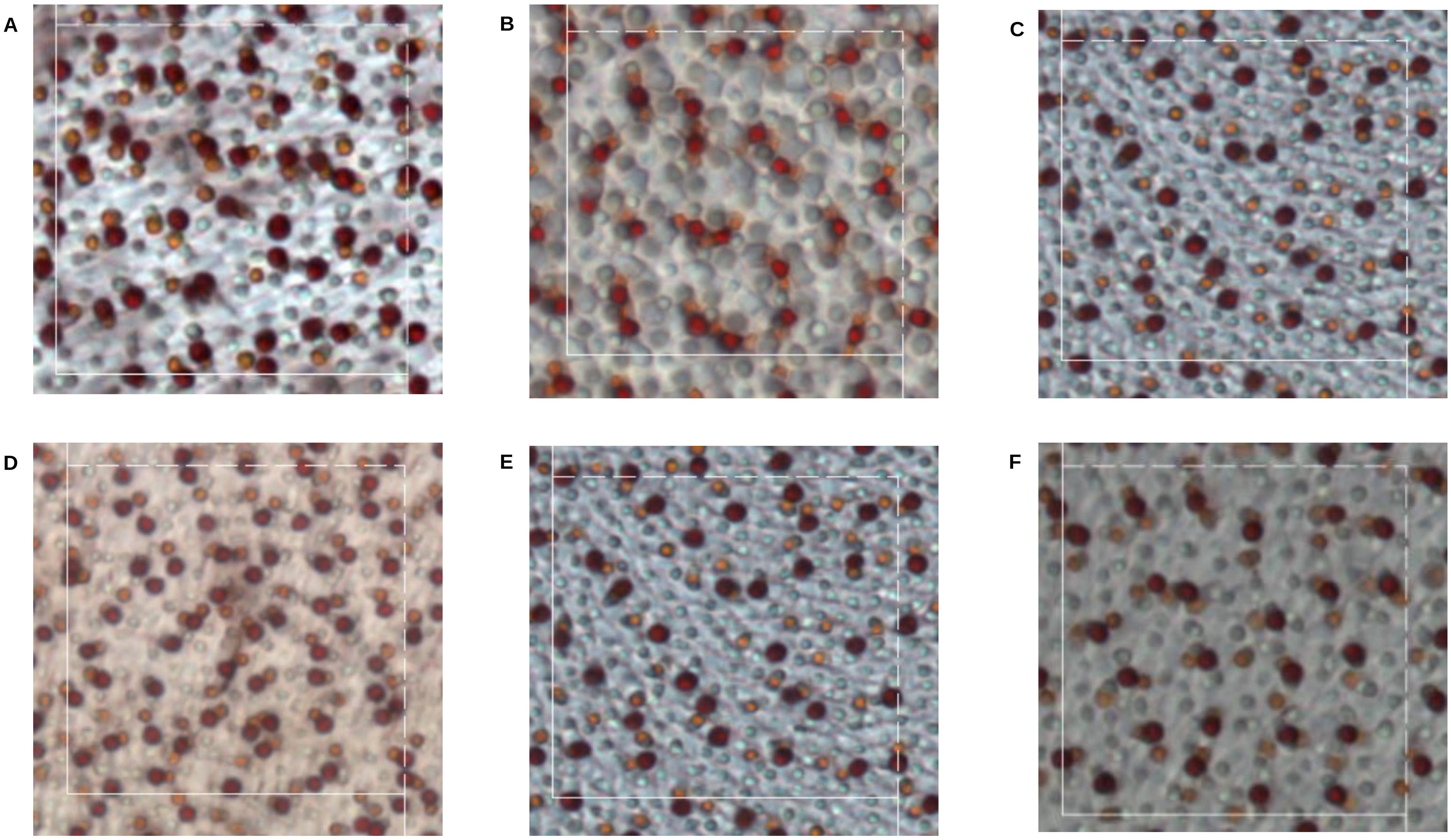

Figure S2. Sample photomicrographs from the center of 6 different individuals to show the distribution of the cone oil droplets. See Table S1 for a description of the different sites, including their distance from the fovea and the cone density counts.

Biology Open • Supplementary information

Figure S3

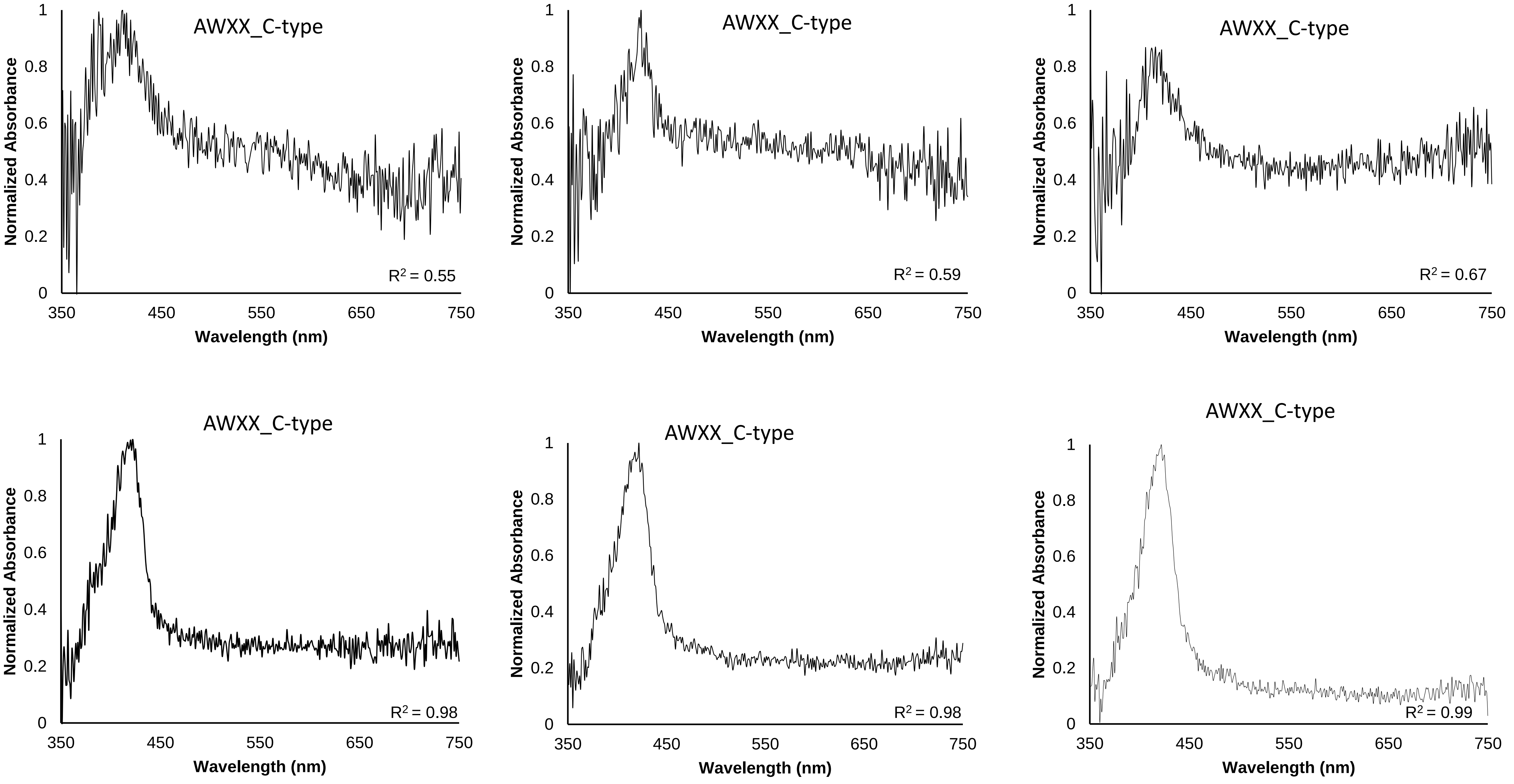

Biology Open • Supplementary information

Figure S4

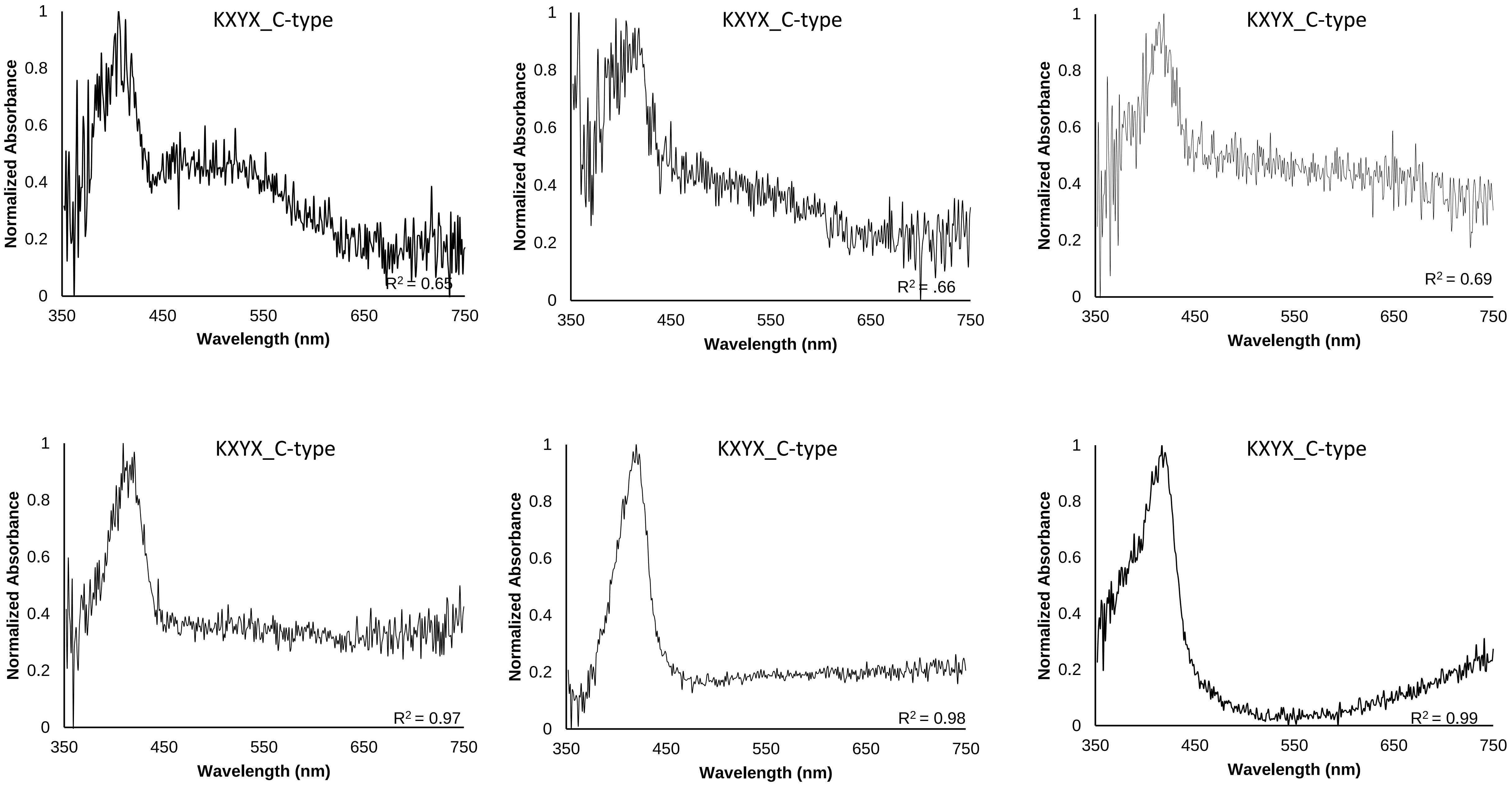
KXYX_C-type
Normalized Absorbance
Wavelength (nm)
R^2 = 0.65
KXYX_C-type
Normalized Absorbance
Wavelength (nm)
R^2 = .66
KXYX_C-type
Normalized Absorbance
Wavelength (nm)
R^2 = 0.69
KXYX_C-type
Normalized Absorbance
Wavelength (nm)
R^2 = 0.97
KXYX_C-type
Normalized Absorbance
Wavelength (nm)
R^2 = 0.98
KXYX_C-type
Normalized Absorbance
Wavelength (nm)
R^2 = 0.99

Figure S5

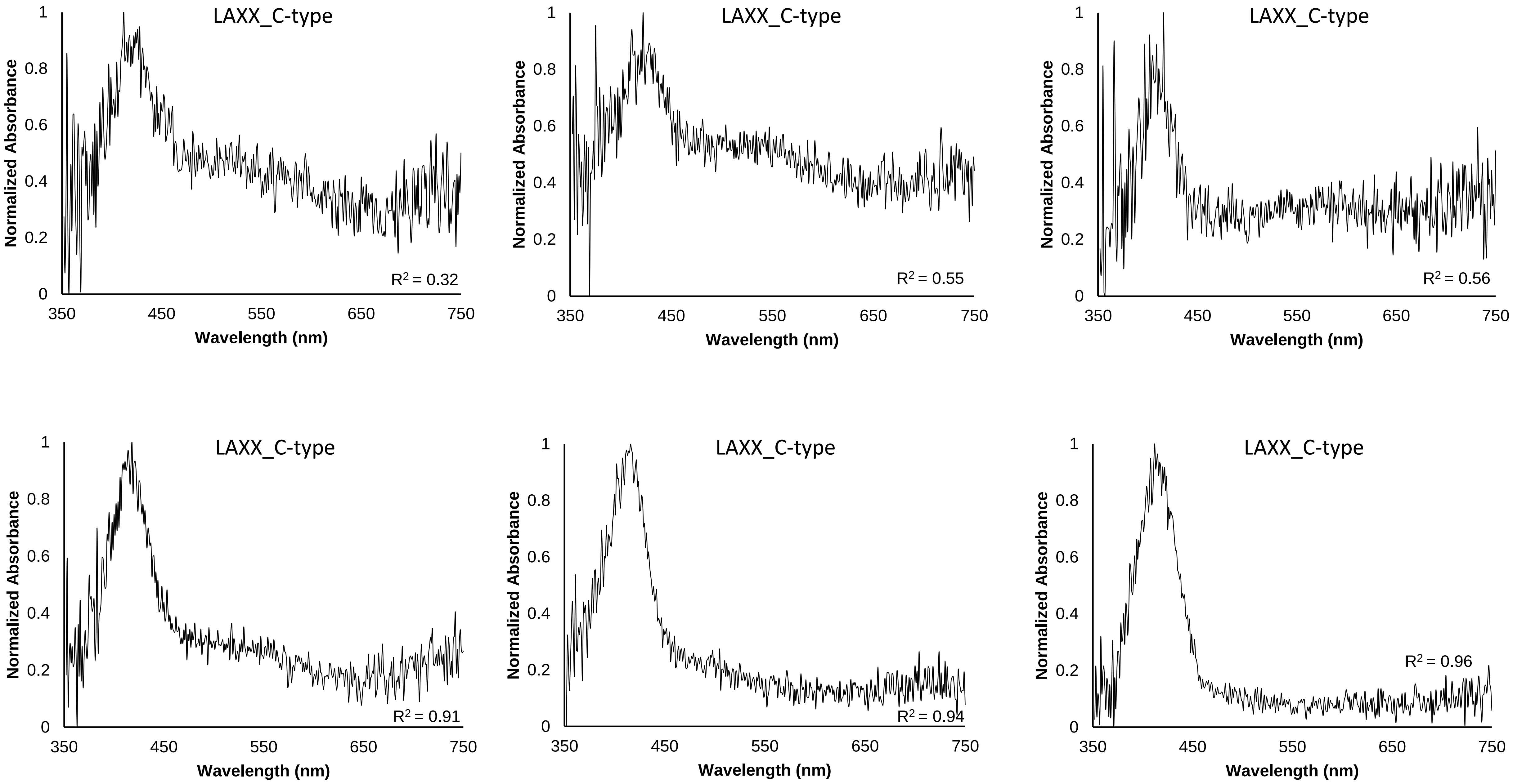

Biology Open • Supplementary information

Figure S6

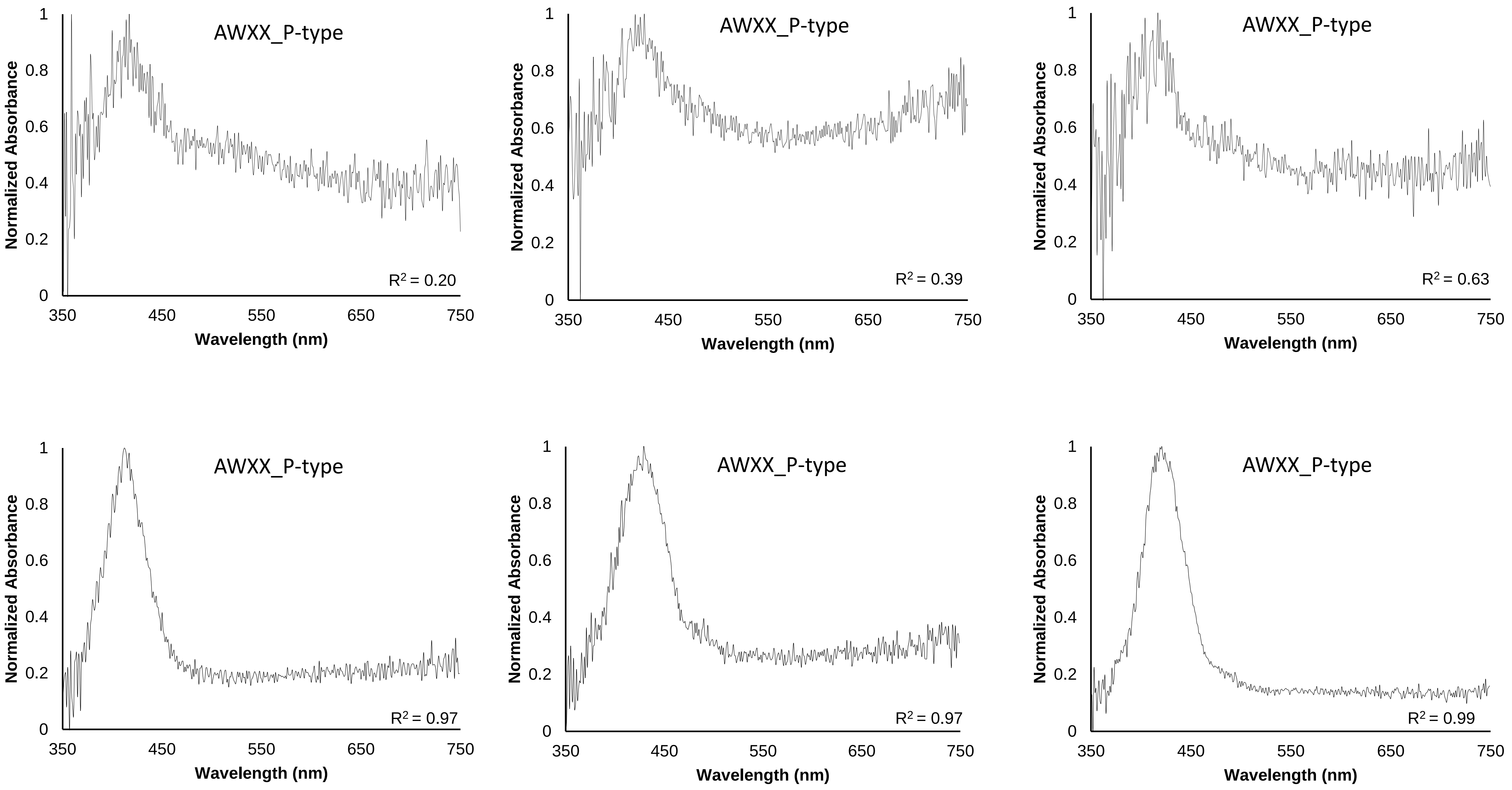

Biology Open • Supplementary information

Figure S7

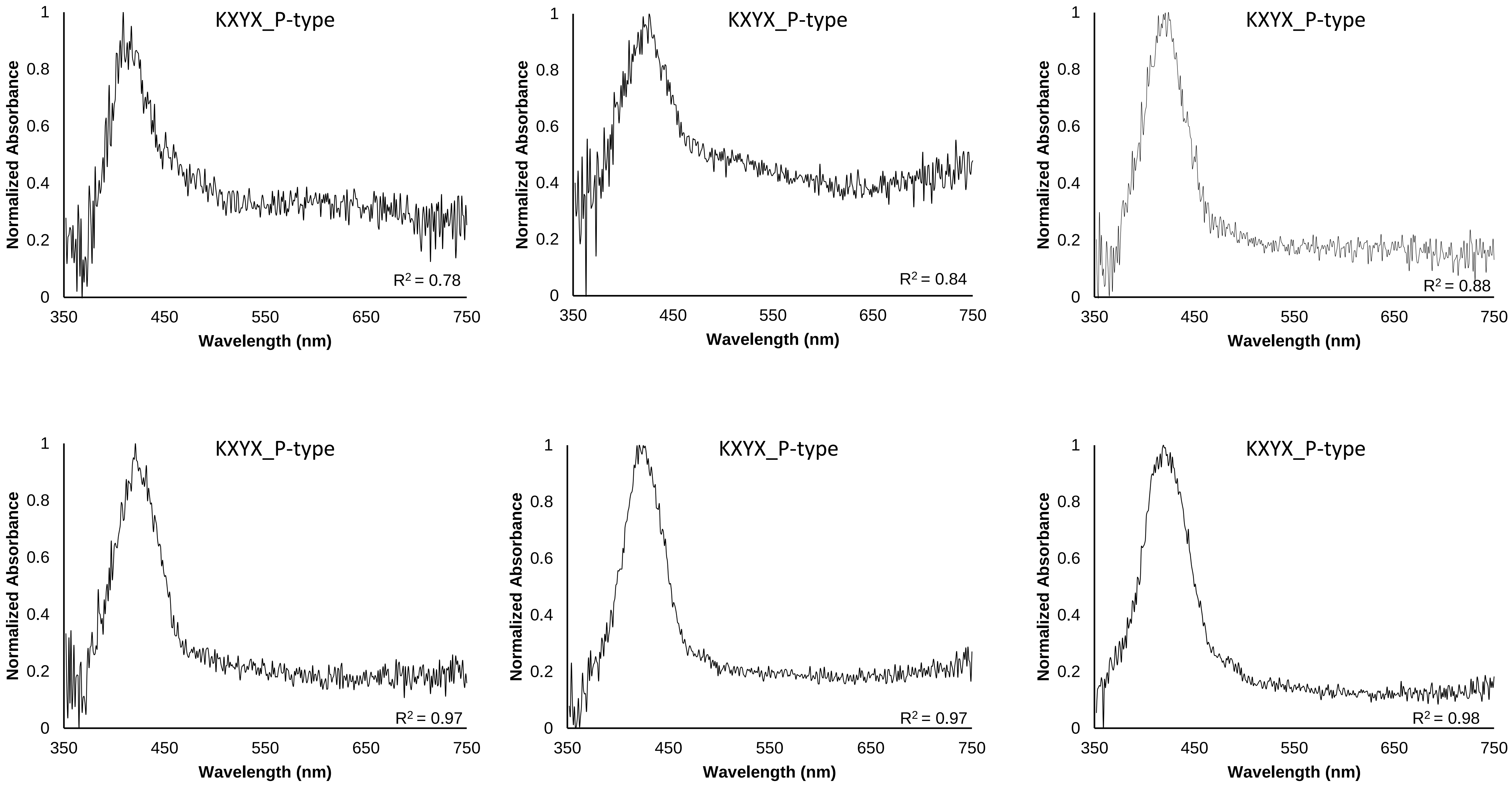
KXYX_P-type
Normalized Absorbance
Wavelength (nm)
R² = 0.78
KXYX_P-type
Normalized Absorbance
Wavelength (nm)
R² = 0.84
KXYX_P-type
Normalized Absorbance
Wavelength (nm)
R² = 0.88
KXYX_P-type
Normalized Absorbance
Wavelength (nm)
R² = 0.97
KXYX_P-type
Normalized Absorbance
Wavelength (nm)
R² = 0.97
KXYX_P-type
Normalized Absorbance
Wavelength (nm)
R² = 0.98

Figure S8

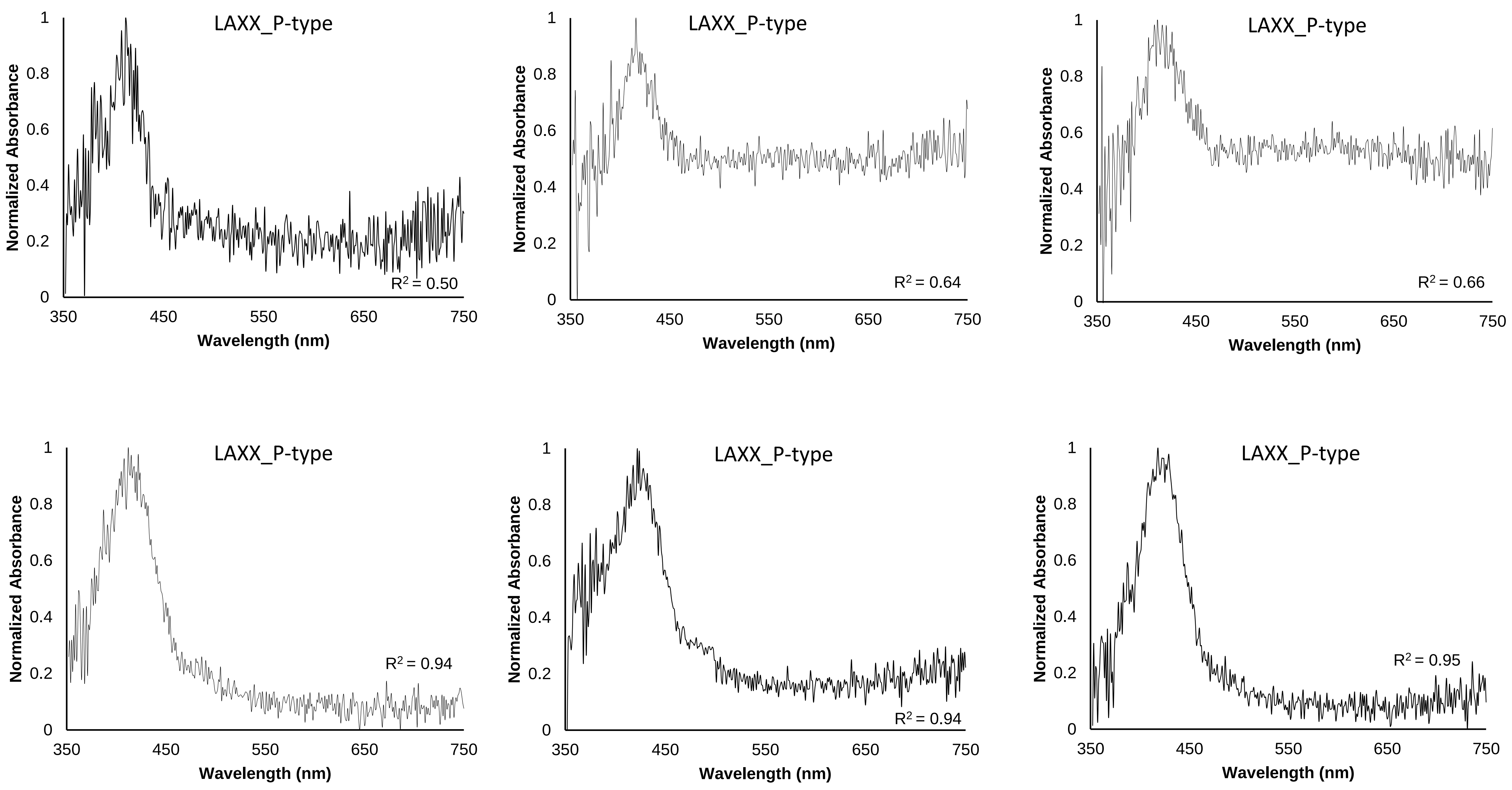

Biology Open • Supplementary information

Figure S9

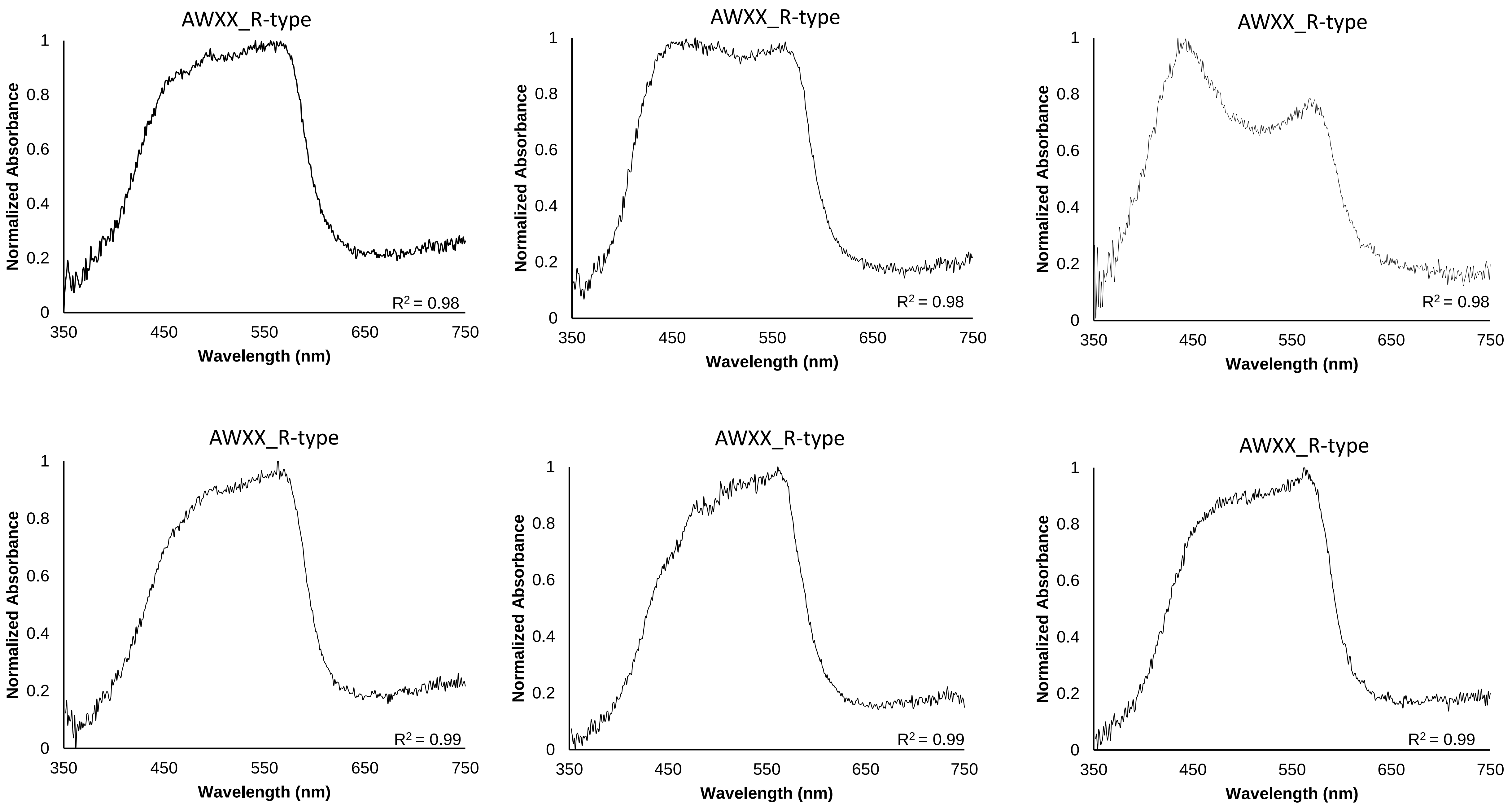

Biology Open • Supplementary information

Figure S10

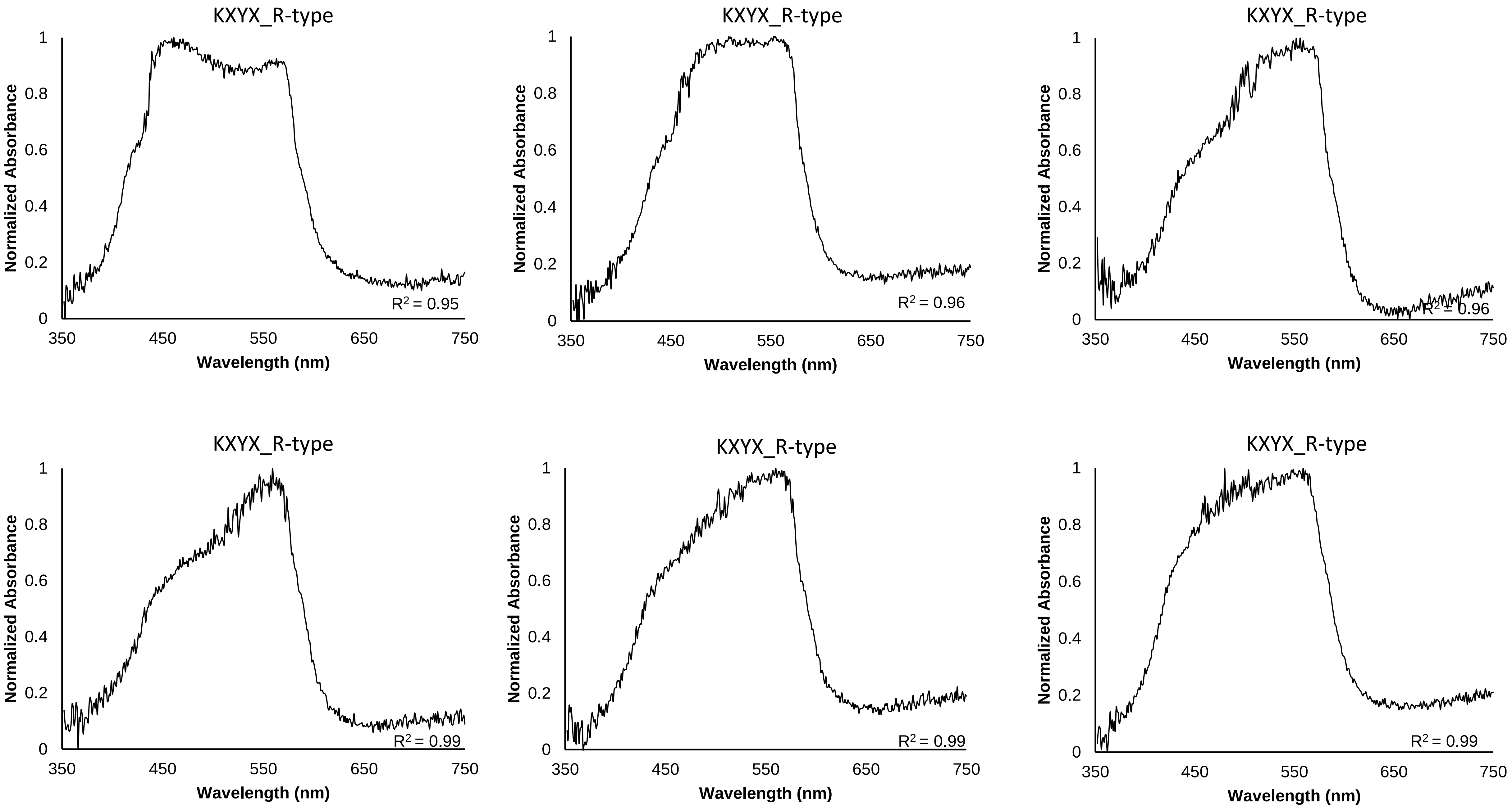

Biology Open • Supplementary information

Figure S11

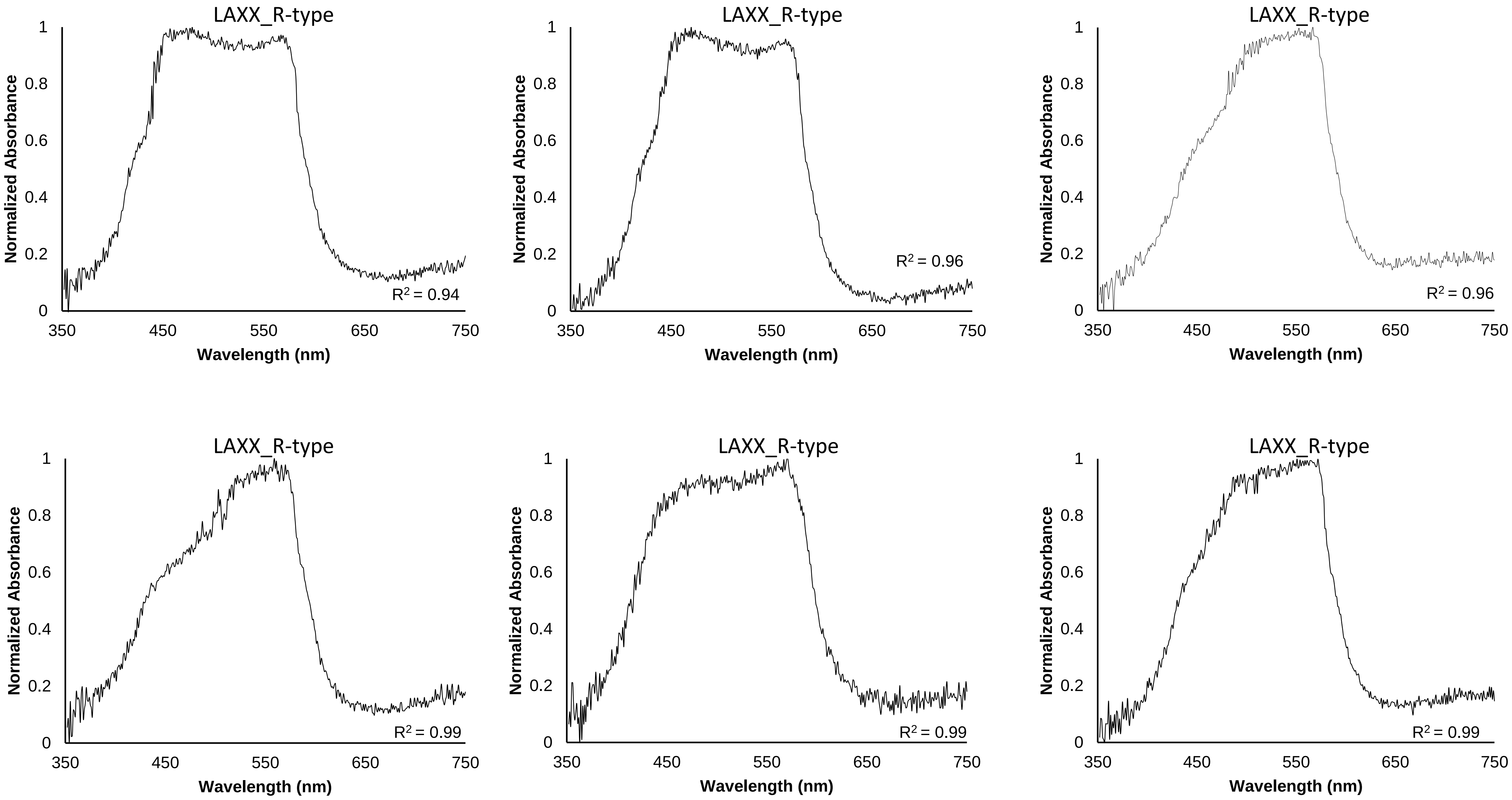
LAXX_R-type
Normalized Absorbance
Wavelength (nm)
R^2 = 0.94
LAXX_R-type
R^2 = 0.96
LAXX_R-type
R^2 = 0.96
LAXX_R-type
R^2 = 0.99
LAXX_R-type
R^2 = 0.99
LAXX_R-type
R^2 = 0.99

Figure S12

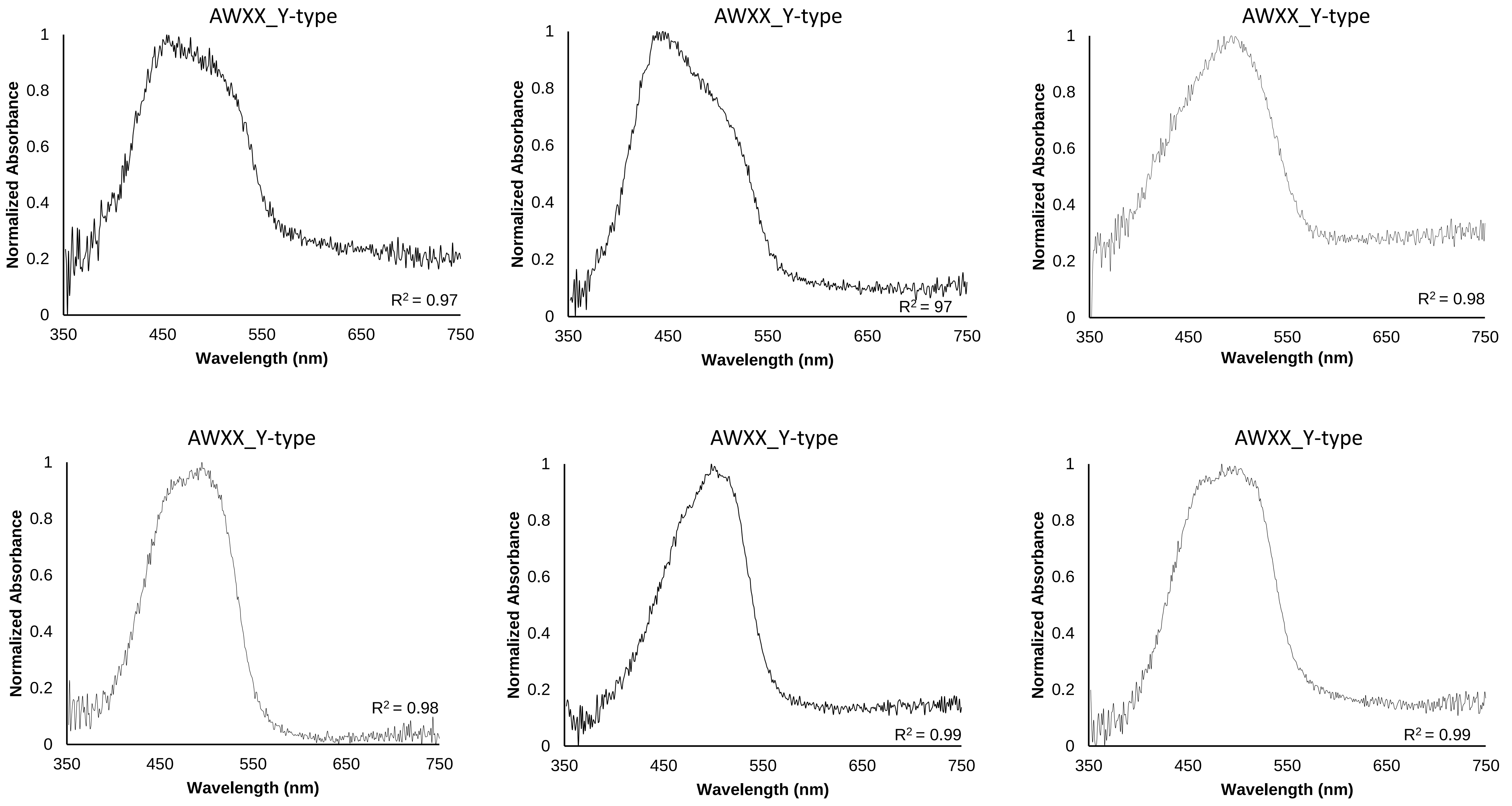

Biology Open • Supplementary information

Figure S13

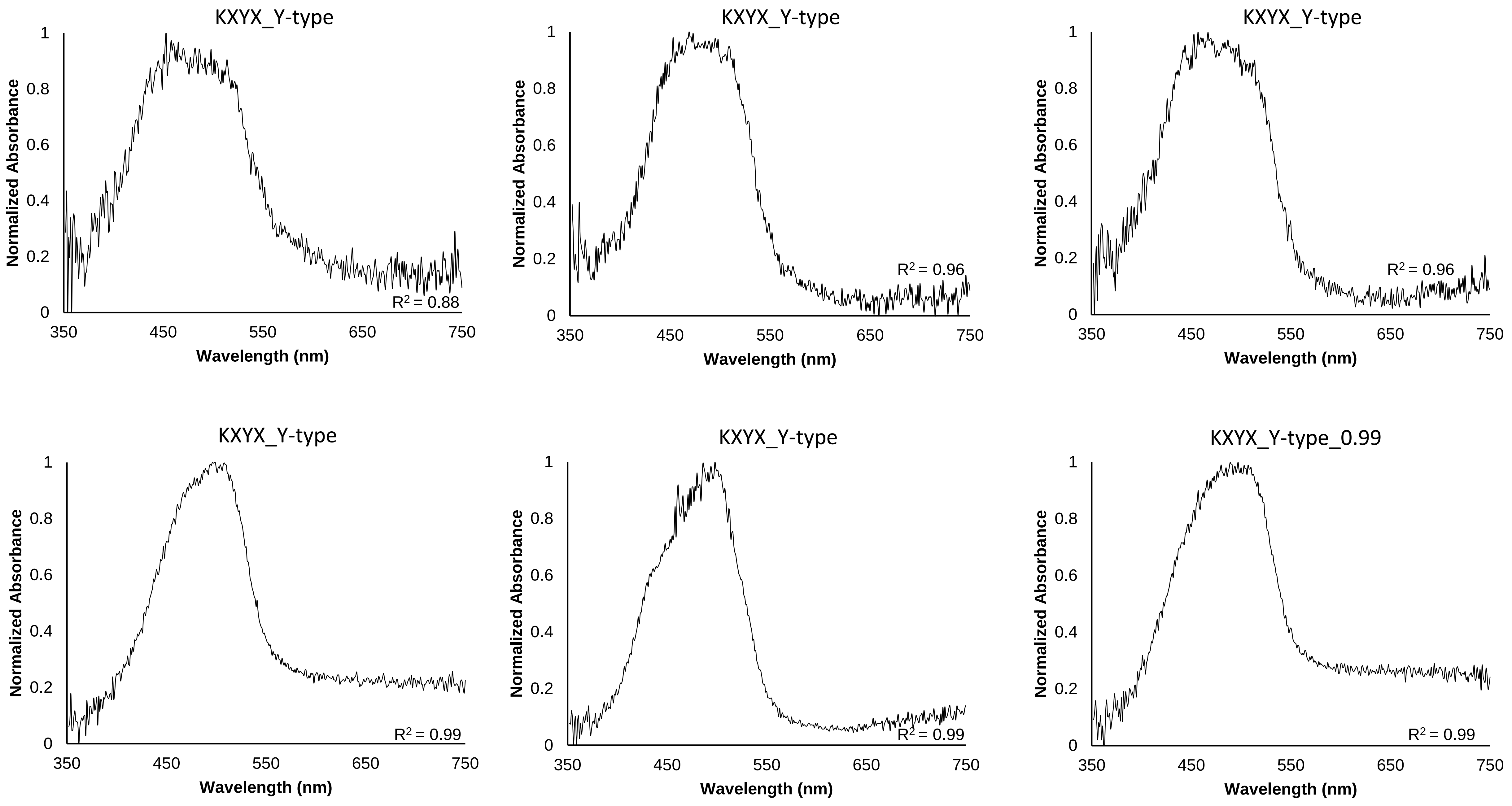

Biology Open • Supplementary information

Figure S14

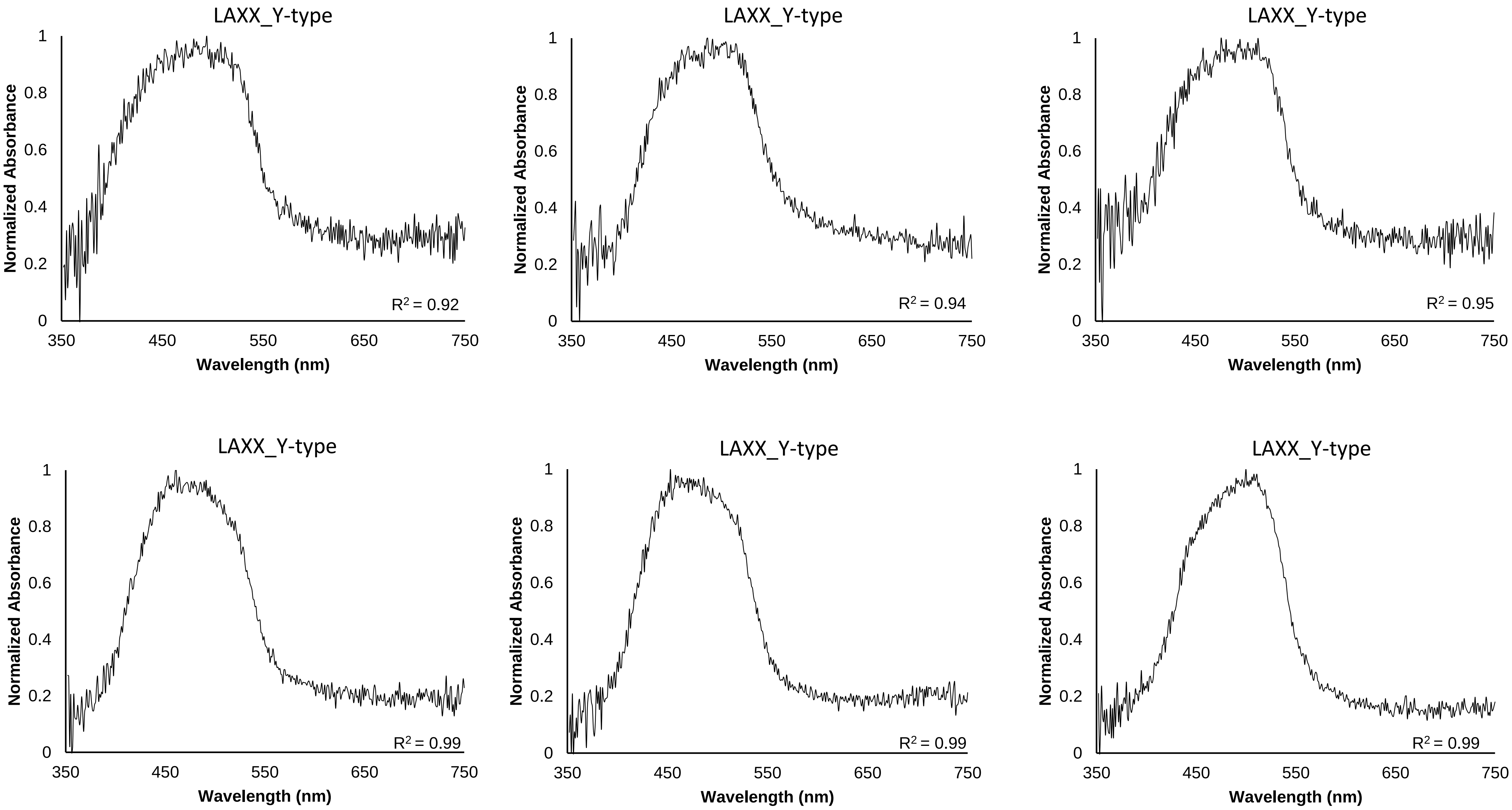

Figure S3-Figure S14. Sample oil droplet absorbance spectra across three individuals, showing 6 different spectra of each oil droplet type (C-type, P-type, R-type, and Y-type) from each individual. Figures S3-S5 show variation in C-type spectra, S6-S8 show P-types, S9-S11 show R types, and S12-S14 show Y types. For each individual and oil droplet type we show the three spectra with the lowest R-squared values and the three spectra with the highest R-squared values to showcase the variation in spectra. Any spectra that had R-squared values below 0.85 were not included in further analyses.

Biology Open • Supplementary information

Figure S15

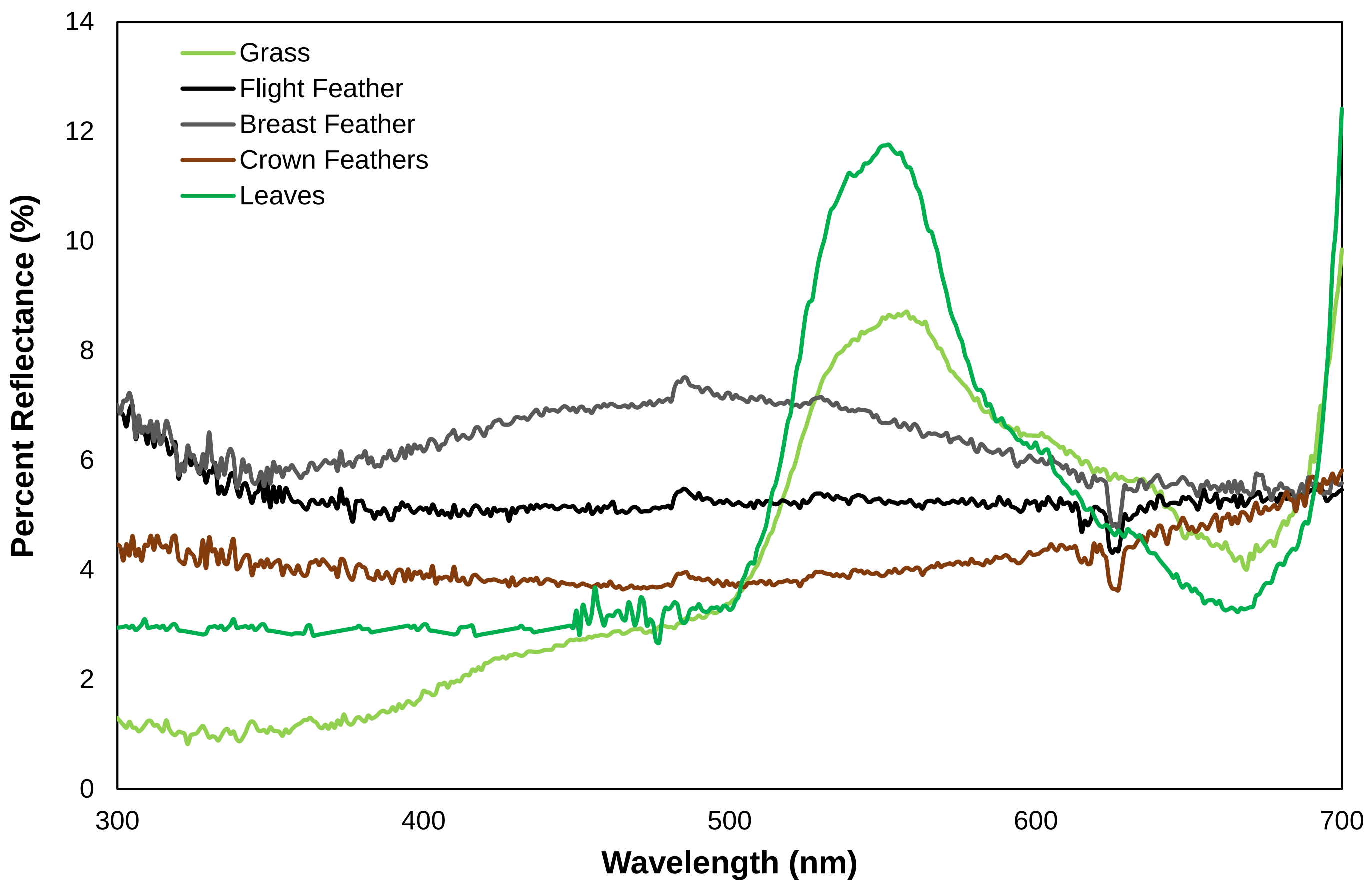

Figure S15. Averaged percent reflectance of the both the grass and leaf backgrounds, and the three feather patches: the crown, flight and breast.

Biology Open • Supplementary information

Figure S16

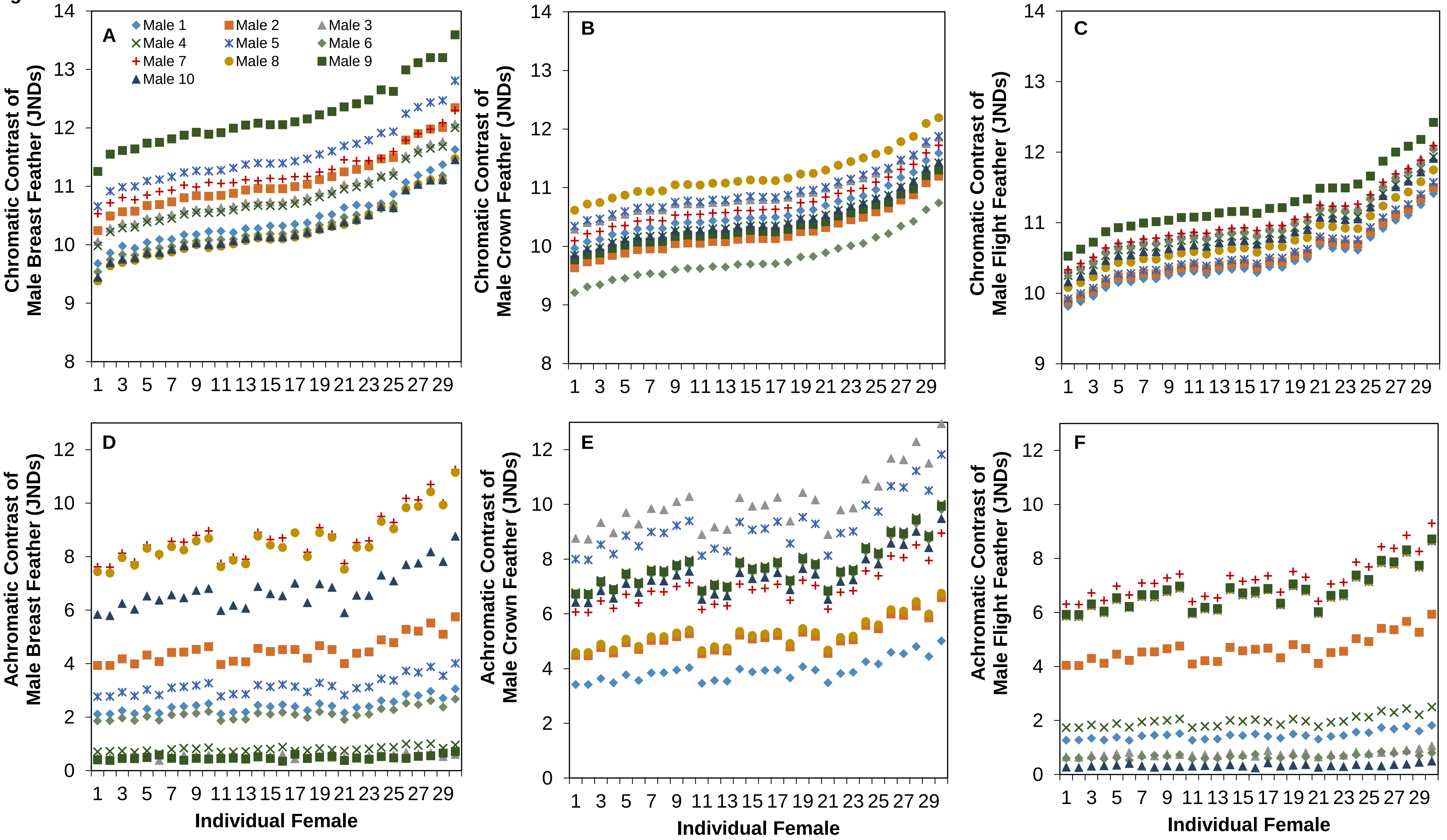

Figure S16. Variation in female perception of chromatic (A-C) and achromatic (D-F) contrasts of male feathers on a grassy background in a sunlit patch. Females varied in their perception of all male feather types: breast feathers (A and D), crown feathers (B and E) and flight feathers (C and F).

Biology Open • Supplementary information

Figure S17

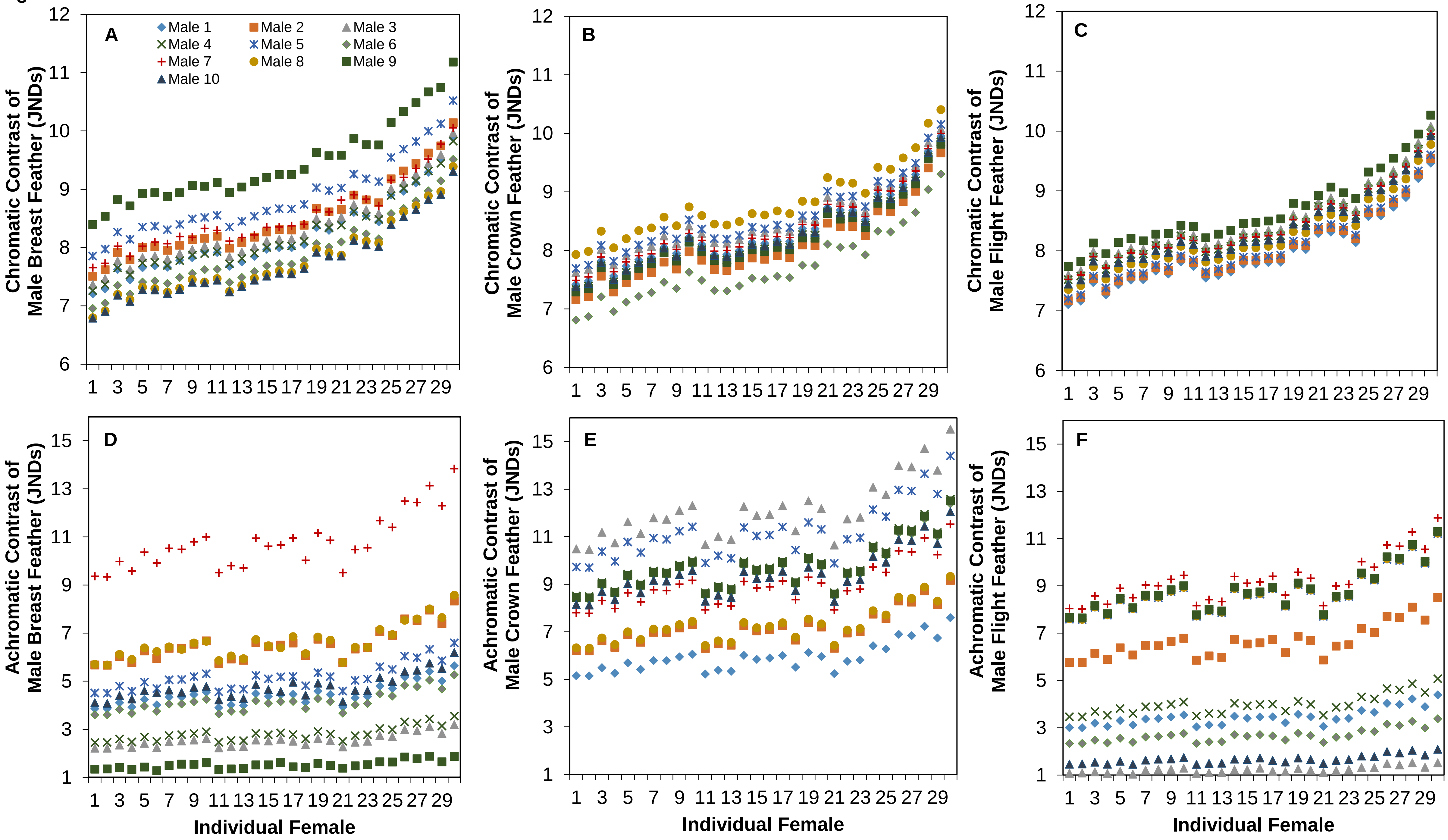

Figure S17. Variation in female perception of chromatic (A-C) and achromatic (D-F) contrasts of male feathers on a leaf background in a sunlit patch. Females varied in their perception of all male feather types: breast feathers (A and D), crown feathers (B and E) and flight feathers (C and F).

Biology Open • Supplementary information

Figure S18

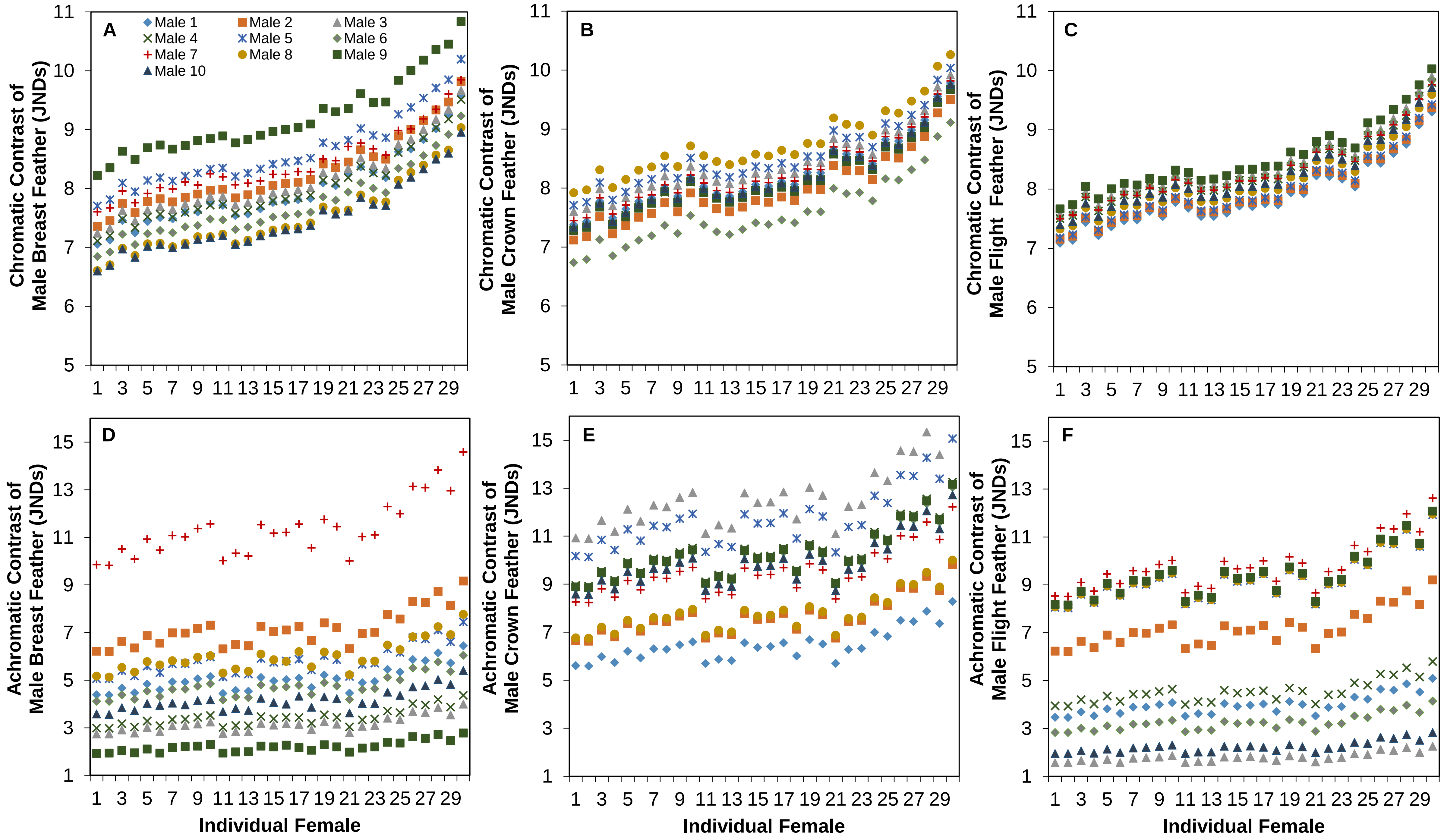

Figure S18. Variation in female perception of chromatic (A-C) and achromatic (D-F) contrasts of male feathers on a leaf background in a shaded patch. Females varied in their perception of all male feather types: breast feathers (A and D), crown feathers (B and E) and flight feathers (C and F).

Biology Open (2017): doi:10.1242/bio.028282: Supplementary information

Figure S19

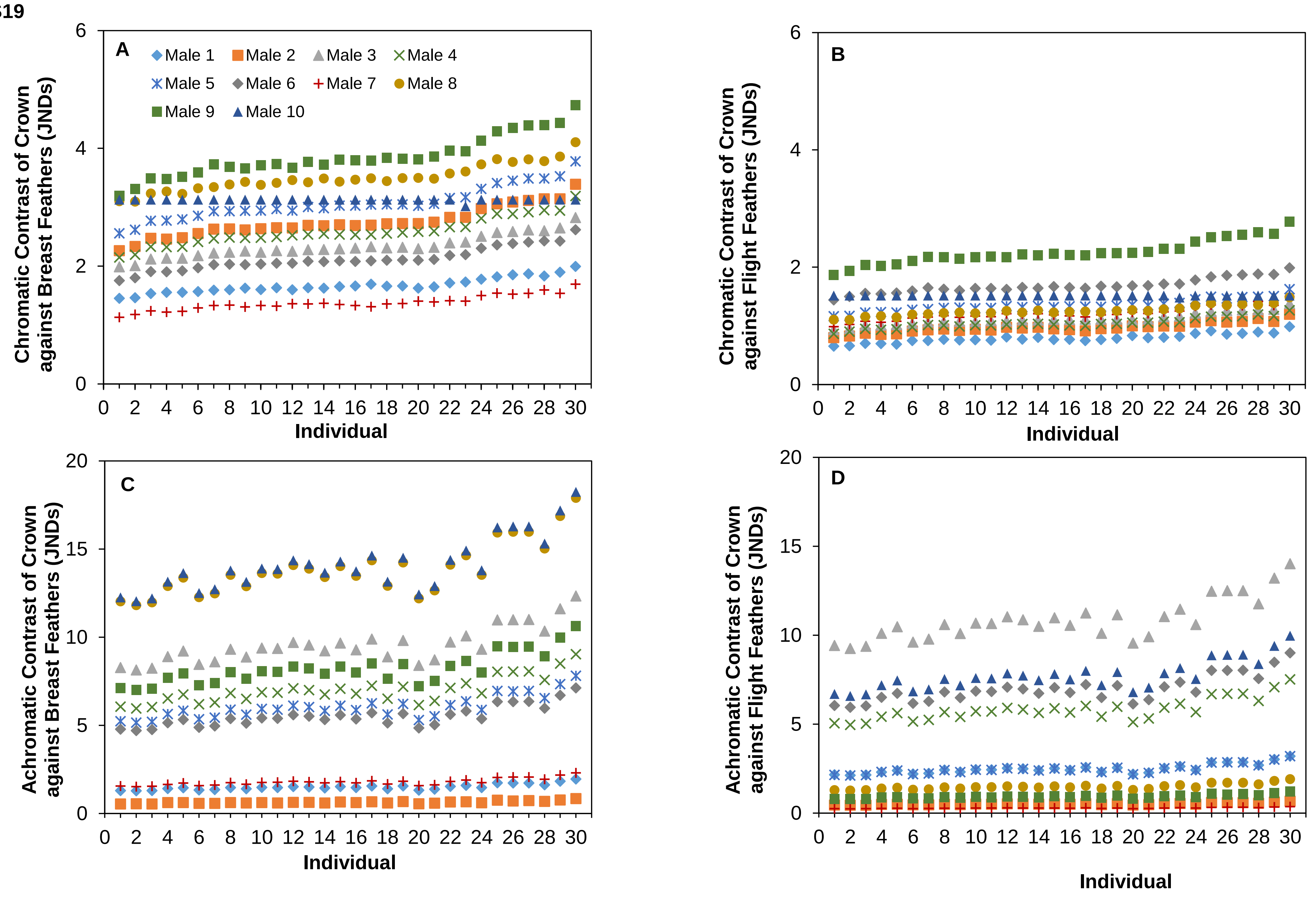

Figure S19. Variation in female perception of chromatic (A and B) and achromatic (C and D) contrasts of male crown feathers either against the breast feathers (A and C) or flight feathers (B and D). These figures represent perception using sunlit patch ambient lighting. Females varied in their perception of all male crown patches.

Biology Open • Supplementary information

Table S1. Description of the six pictures in Figure S2 showing a representative site from near the fovea in six different individuals. This graph details the eccentricity, the distance the picture was from the fovea, the density of the total number of cones at the site as well as the LWS, MWS, UVS, SWS, and double cone densities.

Bird	Eccentricity (μm)	Cones per mm					
		TOTAL	LWS	MWS	UVS	SWS	Double
A	787.809	69,600	10,400	15,200	4,400	12,400	27,200
B	342.357	91,200	16,400	20,800	2,000	15,600	36,400
C	793.711	104,400	12,400	18,800	6,400	22,000	44,800
D	535.027	128,000	23,600	23,200	7,200	22,400	51,600
E	710.286	96,400	18,000	19,200	4,800	15,200	39,200
F	742.113	81,200	10,000	16,800	5,200	14,800	34,400

Biology Open • Supplementary information

Table S2. Data used to model chromatic and achromatic contrast for each individual female (1-30). Cone type ratios, or relative densities, oil droplet absorbance values, and oil droplet Bmid values.

		Cone type ratios			Oil droplet Lambda Cuts				Oil droplet Bmids			
Bird	SWS	MWS	LWS	Double Cone	C Type	Y Type	R Type	P Type	C Type	Y Type	R Type	P Type
1	2.81	3.41	2.13	5.85	422.42	517.17	574.73	426.99	0.04	0.02	0.03	0.03
2	1.83	2.10	1.45	3.95	414.84	508.98	570.81	420.91	0.03	0.02	0.03	0.02
3	2.72	2.79	3.06	6.17	417.28	518.11	575.78	421.16	0.04	0.02	0.03	0.02
4	2.07	2.73	2.60	4.37	413.76	504.76	568.09	421.38	0.04	0.02	0.03	0.02
5	3.41	3.89	3.14	7.78	417.79	509.84	567.04	427.02	0.04	0.02	0.03	0.03
6	3.16	3.70	3.24	6.97	417.62	511.50	565.52	424.41	0.04	0.02	0.02	0.02
7	2.44	2.77	2.15	5.37	417.51	518.40	571.69	423.25	0.03	0.02	0.03	0.03
8	2.65	3.16	2.06	4.96	414.24	512.68	570.08	421.74	0.04	0.02	0.03	0.02
9	2.21	2.50	1.71	4.51	413.59	506.97	571.92	422.53	0.03	0.02	0.03	0.02
10	2.25	2.36	2.07	5.26	422.10	517.50	575.28	425.04	0.04	0.02	0.03	0.03
11	1.91	3.47	2.54	4.07	419.12	513.46	570.90	427.71	0.04	0.02	0.03	0.03
12	2.01	2.62	1.97	4.11	420.49	514.39	572.89	424.44	0.04	0.02	0.03	0.03
13	2.68	3.12	2.55	5.00	422.04	515.96	575.52	428.68	0.03	0.02	0.03	0.03
14	2.03	2.80	2.22	4.91	422.35	509.22	572.51	431.07	0.03	0.02	0.02	0.03
15	3.11	3.33	3.01	6.98	421.04	517.37	569.64	430.58	0.04	0.02	0.03	0.03
16	2.48	2.65	2.50	5.44	417.14	507.57	570.31	421.98	0.03	0.02	0.03	0.02
17	2.93	2.84	2.00	5.63	417.47	510.92	569.49	422.44	0.03	0.02	0.03	0.02
18	2.43	2.59	2.28	4.55	415.65	513.20	570.31	422.27	0.04	0.02	0.03	0.02
19	2.32	2.86	2.34	5.52	416.74	507.77	568.63	417.21	0.03	0.02	0.02	0.03
20	2.05	2.09	1.88	4.17	419.24	514.52	569.88	423.87	0.04	0.02	0.03	0.03
21	2.31	2.64	2.16	5.06	419.28	513.13	570.31	429.48	0.03	0.02	0.03	0.02
22	3.50	4.55	3.55	8.74	416.75	513.58	571.32	420.55	0.04	0.02	0.03	0.03
23	2.21	2.16	1.77	4.88	417.02	517.40	570.90	423.00	0.04	0.02	0.03	0.03
24	2.16	2.48	1.86	4.54	416.12	509.08	566.47	416.20	0.03	0.02	0.02	0.03
25	2.02	2.48	2.13	5.00	418.67	510.20	572.44	424.83	0.04	0.02	0.03	0.03
26	1.95	2.92	2.04	5.43	419.92	510.74	575.88	427.72	0.03	0.02	0.03	0.03
27	1.98	2.55	2.54	4.26	414.65	510.94	565.08	422.79	0.03	0.02	0.02	0.02
28	2.08	2.66	2.22	5.08	420.78	514.72	570.75	425.83	0.04	0.02	0.03	0.03
29	2.74	4.59	3.07	6.92	415.71	513.26	569.79	420.54	0.04	0.02	0.03	0.03
30	1.93	2.14	1.97	3.92	416.99	508.98	568.78	422.60	0.04	0.02	0.03	0.02
